# Mapping of QTLs for morphophysiological and yield traits under water-deficit stress and well-watered conditions in maize

**DOI:** 10.3389/fpls.2023.1124619

**Published:** 2023-05-08

**Authors:** Basudeb Sarkar, Yellisetty Varalaxmi, Maddi Vanaja, Nakka RaviKumar, Mathyam Prabhakar, Sushil Kumar Yadav, Mandapaka Maheswari, Vinod Kumar Singh

**Affiliations:** Division of Crop Sciences, Indian Council of Agricultural Research (ICAR)—Central Research Institute for Dryland Agriculture, Hyderabad, Telangana, India

**Keywords:** drought, physiological and yield traits, SNPs, QTLs, candidate genes

## Abstract

Maize productivity is significantly impacted by drought; therefore, improvement of drought tolerance is a critical goal in maize breeding. To achieve this, a better understanding of the genetic basis of drought tolerance is necessary. Our study aimed to identify genomic regions associated with drought tolerance-related traits by phenotyping a mapping population of recombinant inbred lines (RILs) for two seasons under well-watered (WW) and water-deficit (WD) conditions. We also used single nucleotide polymorphism (SNP) genotyping through genotyping-by-sequencing to map these regions and attempted to identify candidate genes responsible for the observed phenotypic variation. Phenotyping of the RILs population revealed significant variability in most of the traits, with normal frequency distributions, indicating their polygenic nature. We generated a linkage map using 1,241 polymorphic SNPs distributed over 10 chromosomes (chrs), covering a total genetic distance of 5,471.55 cM. We identified 27 quantitative trait loci (QTLs) associated with various morphophysiological and yield-related traits, with 13 QTLs identified under WW conditions and 12 under WD conditions. We found one common major QTL (*qCW2–1*) for cob weight and a minor QTL (*qCH1–1*) for cob height that were consistently identified under both water regimes. We also detected one major and one minor QTL for the Normalized Difference Vegetation Index (NDVI) trait under WD conditions on chr 2, bin 2.10. Furthermore, we identified one major QTL (*qCH1–2*) and one minor QTL (*qCH1–1*) on chr 1 that were located at different genomic positions to those identified in earlier studies. We found co-localized QTLs for stomatal conductance and grain yield on chr 6 (*qg_s_6–2* and *qGY6–1*), while co-localized QTLs for stomatal conductance and transpiration rate were identified on chr 7 (*qg_s_7–1* and *qTR7–1*). We also attempted to identify the candidate genes responsible for the observed phenotypic variation; our analysis revealed that the major candidate genes associated with QTLs detected under water deficit conditions were related to growth and development, senescence, abscisic acid (ABA) signaling, signal transduction, and transporter activity in stress tolerance. The QTL regions identified in this study may be useful in designing markers that can be utilized in marker-assisted selection breeding. In addition, the putative candidate genes can be isolated and functionally characterized so that their role in imparting drought tolerance can be more fully understood.

## 1 Introduction

Maize is a widely consumed staple food and is also used for feed and as an industrial material. However, drought stress has become a major challenge to its productivity, particularly during the anthesis-silking and grain-filling stages (Lobell et al., 2014; Liu and Qin, 2021). Improving drought tolerance in maize is a complex task due to the polygenic nature of this trait and a large amount of genotype × environment interaction (Shinozaki and Yamaguchi-Shinozaki, 2007; Xue et al., 2013; Thirunavukkarasu et al., 2014). Conventional breeding has mainly improved grain yield (GY, g/plant) in favorable environments, and has not done so in drought-prone areas. To address these challenges, a combination of different breeding strategies and the use of genomic tools is necessary. The identification of quantitative trait loci (QTLs) and candidate genes, along with the use of marker-assisted selection in breeding, is critical for this process (Lebreton et al., 1995; Simko et al., 1997; Collins et al., 2008). To improve plant tolerance to drought stress, it is essential to have access to genotypic and phenotypic data, which can be continuously analyzed to gain a better understanding of plant responses (Ribaut et al., 2009). Drought stress can lead to a range of morphophysiological and biochemical changes in plants, such as decreased leaf water content and photosynthesis levels, as well as altered metabolism. These changes can result in reduced plant height, cob weight, biomass, and grain yield (Tester and Langridge, 2010). Linkage and association mapping using next-generation sequencing (NGS) technologies is becoming increasingly popular in the identification of QTLs for complex traits, such as drought tolerance, which is essential for marker-assisted selection (MAS) in breeding. Biparental mapping populations are typically used in QTL identification, in which genotypes with contrasting traits are crossed to produce recombinant inbred lines (RILs), followed by multiple generations of selfing. Through QTL mapping, chromosomal fragments linked with the trait of interest can be identified.

Previously, genetic linkage maps were created using PCR-based markers, such as random amplified polymorphic DNA markers (RAPDs) and simple sequence repeats (SSRs), as well as non-PCR-based markers, such as restriction fragment length polymorphisms (RFLPs). However, rapid advancements in sequencing technology have led to the use of single nucleotide polymorphisms (SNPs) for the development of high-resolution linkage maps (Elshire et al., 2011). These developments in genomics have enabled the mapping of genomic regions associated with drought tolerance through QTLs and association mapping. Several major QTLs associated with drought stress tolerance in maize have been reported in studies by Sanguineti et al. (1999); Malosetti et al. (2008); Messmer et al. (2009); Messmer et al. (2011), Almeida et al. (2013); Liu and Qin (2021), and Leng et al. (2022). In a meta-analysis by Chen et al. (2017), in which 33 published studies of yield-related traits in maize were analyzed, 76 meta-QTLs were identified out of 999 QTLs across the maize genome, although these were reported for normal growth conditions. In a recent review, Liu and Qin (2021) highlighted the progress that has been made in the genetic dissection of drought tolerance in maize at different phenophases of the crop through linkage mapping and association mapping, using various molecular markers including RFLPs, SSRs, and SNPs. In QTL mapping studies, QTLs can be categorized as either ‘constitutive’ (Collins et al., 2008; that is, the same QTLs are detected in different environments) or ‘adaptive’ (that is, QTLs are detected only in specific environments; Almeida et al., 2013). Identification of constitutive or adaptive QTLs can provide valuable insights into ways of improving field-level stress tolerance. Co-localized QTLs for different traits under stress can help in determining whether a particular trait is constitutive or adaptive and in determining its role in improving field-level drought tolerance. This information is important for the identification and selection of appropriate breeding strategies to develop drought-tolerant maize varieties.

Maize is considered a model crop for research in plant genetics due to the availability of a vast amount of omics data (Wallace et al, 2014). The first release of the maize B73 reference genome (Schnable et al., 2009) led to the development of several omics datasets, including DNA resequencing, transcriptomic, metabolomic, and proteomic data (Gore et al., 2009; Chia et al., 2012; Jiao et al., 2012; Li et al., 2013; Wen et al., 2014; Walley et al., 2016; Wen et al., 2018; Jiang et al., 2019; Liu et al., 2020; Wang et al., 2020). Recently, pan-maize gene sets and a pan-*Zea* genome map have been constructed to aid in genetic improvement (Hufford et al., 2021; Gui et al., 2022), and population-level transcripts of diverse lines are also available (Hirsch et al., 2014; Jin et al., 2016). With the wealth of whole-genome sequence data available, mining for candidate genes responsible for phenotypic variation could provide valuable insights into the molecular mechanisms of drought tolerance in maize.

Against this background, the present study aimed to map, through linkage mapping analysis using a subset of maize RILs, the genomic regions that are associated with drought tolerance-related morphophysiological and yield traits, by phenotyping under both well-watered (WW) and water-deficit (WD) conditions and genotyping *via* high-throughput SNP sequencing. An additional aim was the identification of both major and minor effect QTLs and associated candidate genes.

## 2 Materials and methods

### 2.1 Plant material

Contrasting genotypes for drought tolerance were identified based on multi-year evaluation of genotypes for various morphophysiological traits (Maheswari et al., 2016). The drought-tolerant genotype SNJ201126 and susceptible genotype HKI161 were used for development of a mapping population following the single cob method. Initial biparental crossing between tolerant and susceptible genotypes, was conducted during the rainy season of the year 2014. Subsequently, the F_1_ generation was self-pollinated for nine generations to develop a mapping population consisting of 264 single-plant progenies.

### 2.2 Phenotyping for morphophysiological and yield-related traits

The RIL populations, consisting of 264 single-plant progenies and their parents, were planted in a single-row plot of 2.5 m, with 60 cm spacing between rows and 25 cm between plants. Separate experiments were conducted under both WW and WD conditions, following a randomized complete block design (RCBD) with three replications. In the experiment under WW conditions, populations were grown under normal growth conditions until maturity, with irrigation whenever required. However, in the experiment under WD conditions, irrigation was provided only up to the vegetative stage, i.e., 45 days after sowing (DAS); this was followed by imposition of a water deficit for a period of 10 days in order to expose plants to drought stress, which coincided with the anthesis-silking interval (ASI). These two sets of experiments with different treatments were repeated for two seasons: specifically, the rainy season of 2018 and the post-rainy season 2018–19. The experiments were carried out with appropriate plant protection measures in place, and in accordance with recommended practices for growing healthy crop. During the 2018 rainy season, the weekly average temperature varied between 17.9°C and 31.3°C, with relative humidity of 57.1%–84.5%; during the post-rainy season, the temperatures recorded fell between 11.4°C and 30.4°C, with relative humidity of 40.9%–83%. The total rainfall recorded was 377 mm and 16 mm during the rainy and post-rainy seasons, respectively (**Figure S1**).

Various morphophysiological parameters were recorded under both WW and WD conditions: these consisted of Normalized Difference Vegetation Index (NDVI), net CO_2_ assimilation rate (A_net_), stomatal conductance to water vapor (g_s_), transpiration rate (TR), leaf temperature (LT), and anthesis-silking interval (ASI). Yield-contributing traits (i.e., cob height (CH), cob weight (CW), total biomass (TB), and grain yield (GY) were recorded for three representative plants of each genotype. NDVI was measured using a GreenSeeker^®^ 505 device (Manuel NTech Industries Inc., Ukiah, CA, USA). This device measures the reflected light on the canopy of crops in the 660 nm (red) and 770 nm (near-infrared) bands. The NDVI value for any given point in the image, at a particular phenophase of the crop, is equal to the difference in the intensities of reflected light in the red and infrared range divided by the sum of these intensities. A_net_, g_s_, LT, and TR were measured using the LI-6400 portable photosynthesis system (LI-COR Instruments, Inc., Lincoln, NE, USA).

### 2.3 SNP genotyping

The mean phenotyping data for the mapping population (consisting of 264 single-plant progenies), as evaluated under both WW and WD conditions, were used for a cluster analysis, carried out by the average-linkage distance method using SAS^®^ version 9.3 statistical software (SAS Institute Inc., Cary, NC, USA; Cary, 2011). The cluster analysis using combined mean data on morphophysiological and yield-related traits [i.e., relative water content, canopy temperature depression, quantum yield, Soil Plant Analysis Development (SPAD) chlorophyll meter readings, NDVI, proline content, net CO_2_ assimilation rate, g_s_, TR, LT, plant height, CH, cob length, cob girth, number of kernel rows per cob, number of kernels per row, CW, grain yield, TB per plant, and harvest index] under WD conditions was used to group the mapping population into diverse groups. Specifically, the population was grouped into eight clusters (**Table S1**) based on the average distances between all pairs of cluster members between the clusters. The RIL IDs (264 in total), their corresponding cluster IDs, and the corresponding distances are shown in **Table S1**. A subset of 79 RILs were selected from these eight clusters in such a way as to fully capture the genetic diversity of the mapping population (**Table S2**). The frequency distribution of this subset, when compared with the whole population for different traits, was found to represent the phenotypic variation of the population. Along with this subset of 79 RILs, the parents SNJ201126 and HKI161 (in triplicate) were subjected to SNP genotyping at Bionivid Technology Pvt. Ltd., Bengaluru, India. The Illumina NGS workflow for SNP genotyping was employed, as follows. First, the young leaves of 15-day-old seedlings of each genotype were used for DNA isolation using the DNAeasy Plant Mini Kit (Qiagen, Hilden, Germany). Next, the DNA quality and quantity were determined *via* agarose gel electrophoresis and a NanoDrop spectrophotometer, respectively. For library construction, DNA was fragmented randomly and adapters were ligated to the 5′ and 3′ ends. These fragments were then amplified by PCR and purified from the gel. Clusters were generated by loading the library into a flow cell, where fragments were captured on a lawn of surface-bound oligos complementary to the library adapters. After cluster generation, the templates were sequenced. Sequencing was carried out using Illumina SBS technology, a system that detects single bases as they are incorporated into template strands. As all four reversible terminator-bound dNTPs are present during each sequencing cycle, natural competition minimizes incorporation bias and reduces the raw error rate in comparison to other technologies. This enables highly accurate base-by-base sequencing that virtually eliminates sequence-context-specific errors, even within repetitive sequence regions and homopolymers. Sequencing data were subsequently converted to raw data for analysis. The Illumina sequencer generates raw images utilizing sequencing control software for system control and base calling through an integrated primary analysis software tool called Real-Time Analysis (RTA). The base calls (BCL) binary was converted to FASTQ format using Illumina package bcl2fastq. The total numbers of bases and reads, along with values of GC (%), Q20 (%), and Q30 (%), were calculated for all samples. The raw sequences of genotyping data were deposited in the NCBI database (http://www.ncbi.nlm.nih.gov/sra/PRJNA913688) with accession number PRJNA913688.

### 2.4 Statistical analysis

The phenotyping data of the subset of 79 RILs, which were generated in two seasons under WW and WD conditions, were used for statistical analysis, as the sequencing data were generated for the same set. Individual and combined (i.e., over seasons and treatments) analyses of variance (ANOVAs) were carried out for morphophysiological traits; Pearson’s simple correlation coefficients were also calculated and heritability estimates were made using SAS version 9.3. For combined analyses, the homogeneity of variance component was determined using Bartlett’s test (Bartlett, 1937). Broad-sense heritability was calculated as per the following formula:


$$\text{Broad‐sense heritability}\;{(}^{\;}{\text{H}}^{2})\;=\;\frac{{\text{σ}}^{2}G}{{\text{σ}}^{2}P}$$

where ${\text{σ}}^{2}G$ is the total genotypic variance and ${\text{σ}}^{2}P$ is the total phenotypic variance. Frequency distribution histograms for all traits were generated using Matplotlib tools (Hunter, 2007). Matplotlib is a cross-platform data visualization and graphical plotting library for Python. The Pyplot module was used to generate plots, and the Scipy.stats module (Virtanen et al., 2020) was used to compute and draw histograms of the WW and WD data; evenly spaced points over a specified interval on the *x*-axis were created using numpy.linspace, and the norm of the probability density function is displayed on the plots. The Kolmogorov–Smirnov method (Kolmogorov–Smirnov Test, 2008) was used to test for normality. Descriptive statistics were calculated using SAS, version 9.3.

### 2.5 Bioinformatics analyses

#### 2.5.1 SNP calling and filtering

The raw reads of the sequencing data of 79 RILs and their parents were generated in FASTQ format for all samples and imported into a TASSEL GBS pipeline (Glaubitz et al., 2014; Zhang et al., 2015), implemented in TASSEL version 5.0. Maize genotype B73, Zm-B73-Reference-NAM-5.0-Genome-Assembly–NCBI (https://www.ncbi.nlm.nih.gov/assembly/GCF_902167145.1/) was used as the reference genome. The qualifying filtering steps of the sequence reads were mapped onto the genome using the Burrows–Wheeler Alignment (BWA) tool (Li and Durbin, 2009). The mapped reads were then exported as a sequence alignment map (SAM) file for SNP calling and genotyping (Bradbury et al., 2007). A total of 176 Gb of data was generated for all sequenced samples. To filter the parent’s call, replicates were first merged by ensuring that at least two replicates had observed calls, and the most common allele was taken as the parent call, with the alternate call within replicates of parents taken to reflect genotyping errors. Functional annotation of SNPs was carried out using the Ensembl Plants variant effect predictor (VEP) tool (http://plants.ensembl.org/Zea_mays/Tools/VEP?db=core) with maize reference assembly Zm-B73-REFERENCE-NAM-5.0. The SNPs already reported in the database were clustered into a non-redundant reference SNP cluster and assigned a unique rsID; for SNPs that did not clearly correspond to a clear rsID, an internal ID was given. In addition, SNPs with a minor allele frequency (MAF), i.e., a frequency of< 5%, were filtered out before analysis.

#### 2.5.2 Linkage map construction and QTL analysis

Linkage map construction and QTL analysis were carried out using QTL IciMapping software, version 4.2 (Meng et al., 2015). The SNP data consisting of DNA bases (i.e., A, T, G, or C) were converted into the format recognized by the QTL IciMapping software using the SNP conversion functionality. SNPs showing non-polymorphism in parents or progenies, or missing in one or more of the parents, were removed by this functionality. The datasets thus generated for the RILs population therefore consisted of either one of the parental types (A or B) or missing data. Linkage map construction was carried out using the MAP functionality and comprised three steps: grouping, ordering, and rippling. The grouping of markers was based on anchored marker information and a logarithm of the odds (LOD) threshold score of 2.5 for unanchored markers. The ordering algorithm used was K-optimality by recombination, using the random nearest neighbor (NN) count route (10). The criteria used in rippling were a window size of five and recombination frequency (REC). Finally, the output was used to generate the linkage map. The anchoring and genotypic data generated along with the phenotypic data were used for QTL identification. A total of 1,241 SNPs were finally selected for analysis. The Biparental Populations (BIP) functionality of the software was used to study the association of these SNPs with morphophysiological and yield-related traits. The mapping method used was the inclusive composite interval mapping (ICIM) method for QTL with additive effects (i.e., ICIM-ADD). The mapping parameters set were stepwise scanning by 1 cM, deletion of missing phenotypic data, phenotype on marker variables (PIN) of 0.001, and a LOD threshold of 2.5. The QTL effects were estimated based on LOD, additive effect of identified loci, and percentage of phenotypic variation explained (PVE%). QTLs with PVE% of ≥ 15% and< 15% were regarded as major and minor effect QTLs, respectively. The standard procedure was followed for QTL nomenclature (McCouch, 2008).

The linkage map view package of R (The R Foundation for Statistical Computing, Vienna, Austria; Ouellette et al., 2018) was used to display the QTLs identified under WW and WD conditions, capturing only the regions where QTLs were located. An epistatic analysis was carried out using the IciMappingVer.4.1 EPI epistatic module, with default parameter settings (LOD = 5, step = 1 cM, and stepwise regression probability< 0.0001). The combined phenotyping data from two seasons (i.e., the rainy and post-rainy seasons) and two different environments (i.e., the WW and WD conditions) were used to carry out a joint QTL analysis with additive-by-environment (A-by-E) interactive effects in a multi-environment trial (MET) module of the ICIM method (Meng et al., 2015). The parameters of the QTL analysis were set as follows: LOD threshold: 2.5; 1,000 permutations; step: 1 cM; and PIN: 0.001. The confidence interval (CI) of each QTL was determined by using LOD > 3.

#### 2.5.3 Functional annotation of SNPs

Functional annotation of the selected SNPs was carried out using the VEP tool in Ensembl Plants (https://plants.ensembl.org/Zea_mays/Tools/VEP). The VEP tool analyses the variants and predicts the functional consequences of both known and unknown variations. The reference genome assembly was Zm-B73-REFERENCE-NAM-5.0 for each SNP to identify rs-ID, location, allele, consequence, gene, feature type, biotype, exon/intron, and TREMBL protein IDs were identified.

#### 2.5.4 Identification of candidate genes in the genomic region spanning QTLs for water-deficit stress conditions

The QTL intervals obtained in the linkage map were further studied for the prediction of candidate gene(s) associated with the respective QTLs, using the maize genome sequence available at Ensembl Plants (https://ensembl.gramene.org/Zea_mays/). Marker intervals were mapped for their physical locations and the sequences between the intervals were retrieved using NCBI-BLAST. The numbers and types of genes present in the sequences were also identified. Functional annotation of the genes present within the QTL regions was carried out using the Database for Annotation, Visualization and Integrated Discovery (DAVID) tool for gene functional annotation (https://david.ncifcrf.gov/summary.jsp) and the maize genetics and genomics database (GDB) (https://www.maizegdb.org). The genomic region covering each QTL was further searched for the presence of QTLs reported by genome-wide association studies using the maize genome database (https://jbrowse.maizegdb.org).

## 3 Results

### 3.1 Phenotypic variation in the mapping population

**Figure 1:**
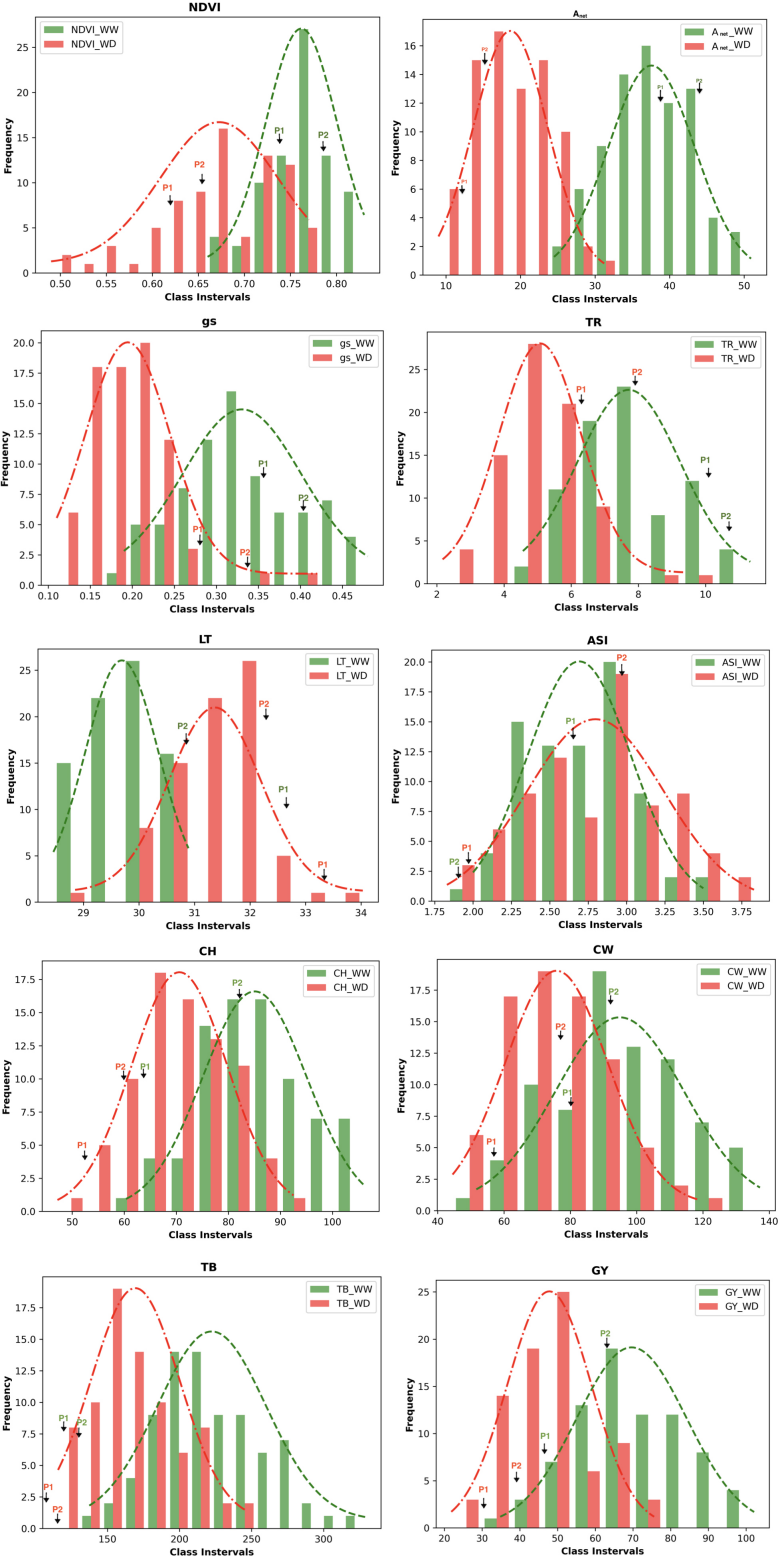
Frequency distribution of the phenotypic data of the RILs population of the various morphophysiological and yield related traits. The values of the parents (P_1_ - HKII61 and P_2_ SNJ201126 are indicated by arrows); NDVI, Normalized difference vegetation index; Anet, Net CO_2_ assimilation rate; g_s_, Stomatal conductance to water vapor; TR, Transpiration rate; LT, Leaf temperature; CH, Cob height; ASI, Anthesis-silking interval; CW, Cob weight; TB, Total biomass; GY,Grain yield/plant; WW, Well-watered; WD, Water-deficit stress.

The population consisted of 79 RILs, which demonstrated wide variation in their morphophysiological and yield-related traits (i.e., NDVI, A_net_, g_s_, TR, LT, ASI, CH, CW, TB, and GY) under WW and WD conditions (**Table S3**). All traits were affected by WD. Histograms of the frequency distributions of these traits in the WW and WD conditions are presented in **Figure 1**. All traits followed a near-normal distribution, with the exception of stomatal conductance, which is positively skewed in the WW condition. Based on the significance values obtained using the Kolmogorov–Smirnov test, the traits A_net_, LT, CH, CW, GY, and TB followed a normal distribution under both under WW and WD conditions, whereas the traits g_s_ and TR followed a normal distribution under the WD condition (**Table S4**). This indicates that the selected RILs captured the genetic variability of the entire population to be utilized for QTL identification. The descriptive statistics of the mapping population under WW and WD conditions for morphophysiological and yield-related traits, including coefficients of variation (CV%), skewness, kurtosis, and heritability, are provided in **Table S5**. Moderate broad-sense heritability (H^2^), ranging between 0.45 and 0.71, was observed for all traits, and a wide range of coefficients of variation was also observed among these traits.

A combined ANOVA over trials across seasons (i.e., rainy vs. post-rainy) and conditions (i.e., WW vs. WD) indicated significant interactions among season, treatment type, and genotype for most traits. For total biomass (TB), all interaction effects were significant except seasons × treatments (**Table S6**). For ASI, the interaction effects for treatments × genotypes and seasons × treatments × genotypes were non-significant. The significant variation by genotype, environment, and their interaction for a number of traits indicated that these traits were influenced by both genetic and environmental factors. A simple correlation coefficient analysis revealed significant positive correlations of NDVI with CH, CW, GY, and TB; A_net_ with g_s_ and TR; g_s_ with TR; CH with TB, GY, and TB; CW with GY and TB; and GY with TB under both WW and WD conditions. In addition, a significant positive correlation of NDVI with LT, and of CH with CW and GY, was observed under WD conditions (**Table S7**).

### 3.2 QTL mapping

**Figure 2:**
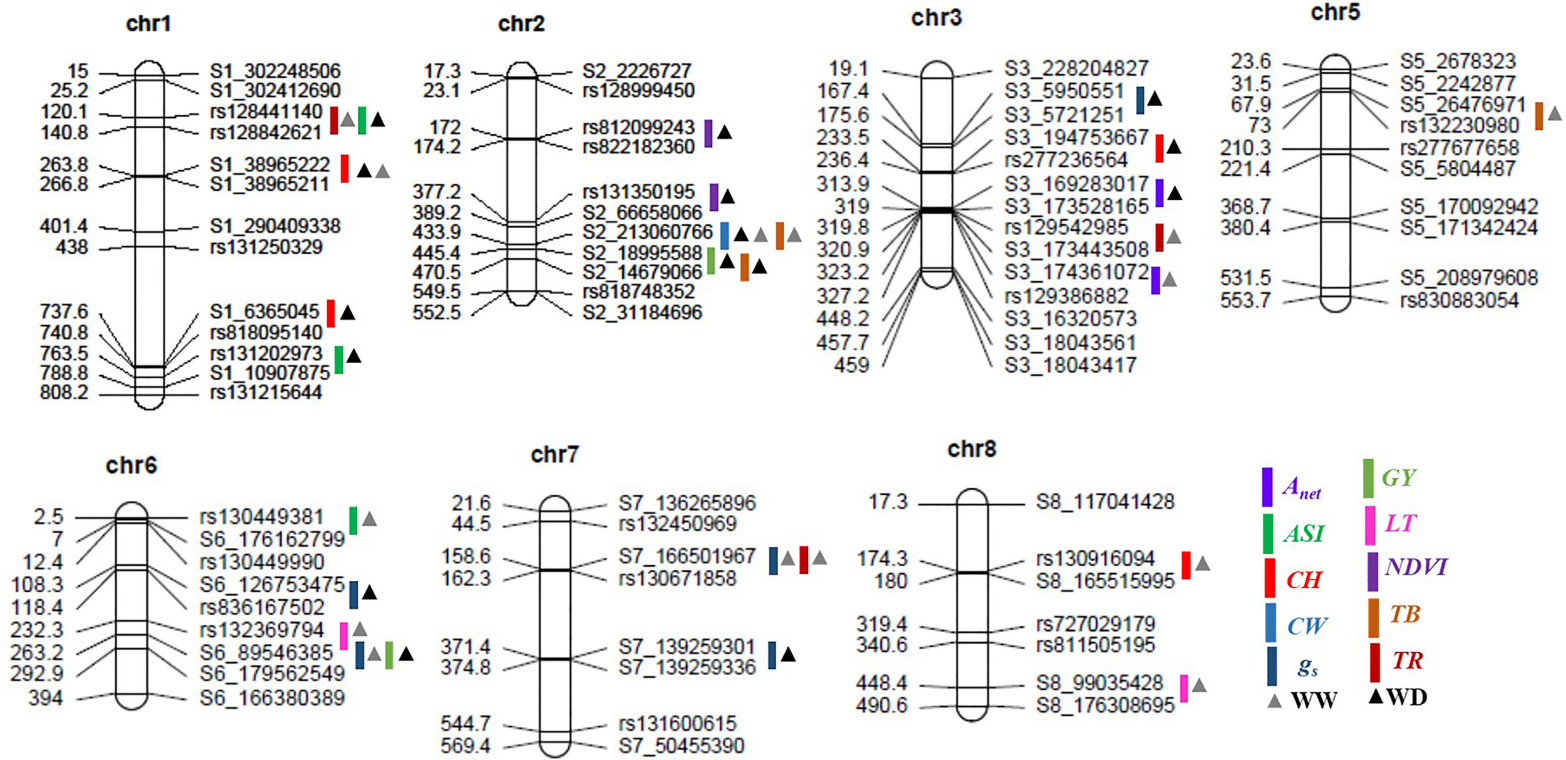
Positions of the quantitative trait loci (QTLs) for various morphophysiological and yield-related traits on seven chromosomes in the recombinant inbred line (RIL) population grown under well-watered and water-deficit conditions. The scaled numbers on the left side of the chromosomes indicate genetic length (cM), with the corresponding markers on the right side. The colored bars and triangles represent the QTLs identified for the various traits and the condition, respectively. A_net_, net CO_2_ assimilation rate; ASI, anthesis-silking interval; CH, cob height; CW, cob weight; g_s_, stomatal conductance to water vapor; GY, grain yield; LT, leaf temperature; NDVI, Normalized Difference Vegetation Index; TB, total biomass; TR, transpiration rate; chr, chromosome; WW, well-watered conditions; WD, water-deficit conditions.

The numbers of raw SNPs and polymorphic SNPs between the parents after filtering with MAF< 5%, along with their distribution in various chromosomes, the number of mapped SNPs in the linkage map, and the average marker interval, are presented in **Table 1**. The largest number of markers (219) was detected on chr 2, and the smallest (65) on chr 10. In the present study, 27 QTLs were identified; of these, 13 were detected under WW conditions, 12 under WD conditions, and two (*qCH1–1* and *qCW2–1*) under both WW and WD conditions (**Table 2**; **Figure 2**). Major and minor QTLs were detected only in chromosomes 1, 2, 3, 5, 6, and 7 (out of the 10 maize chromosomes studied under WW and WD conditions), and no QTLs were detected in chromosomes 4, 8, 9, or 10. Under WD conditions, QTLs for CH (*qCH1–1* and *qCH1–2*) and ASI (*qASI1–1*) and for NDVI, CW, TB, and GY were detected in chromosomes 1 and 2. Similarly, three (*qA_net_3–1, qg_s_3–1, qCH3–1*), two (*qg_s_6–1, qGY6–1*) and one (*qg_s_7–2*) QTLs were detected in chromosomes 3, 6, and 7, respectively, under WD conditions (**Figure 2**). QTLs with PVE% greater than 15% were regarded as major QTLs. Among the 13 QTLs detected under WW conditions, three major QTLs were identified for traits A_net_, g_s_, and TR, with LOD scores ranging from 2.54 to 6.07 and PVE% ranging from 15.86% to 21.47%. Ten minor QTLs were detected for traits LT, TR, g_s_, TB, CH, and ASI, with LOD scores ranging from 2.54 to 3.2 and PVE% ranging from 6.75% to 14.91% (**Table S8**). Among the 12 QTLs detected under WD conditions, three were major QTLs identified for traits NDVI, g_s_, and CH, with LOD scores ranging from 3.49 to 4.45 and PVE% ranging from 15.05% to 18.59%. Nine minor QTLs were detected for traits A_net_, NDVI, ASI, CH, g_s_, TB, and GY, with LOD scores ranging from 2.52 to 3.37 and PVE% ranging from 9.69% to14.25% (**Table 2**). Among these, two QTLs were detected under both WW and WD conditions: these were a minor QTL for the CH trait and a major QTL for the CW trait, located on chromosomes 1 and 2, respectively.

**Table 1:** Numbers of single nucleotide polymorphisms (SNPs) on the 10 chromosomes of maize used for quantitative trait locus (QTL) mapping.

SNPs	Chr 1	Chr 2	Chr 3	Chr 4	Chr 5	Chr 6	Chr 7	Chr 8	Chr 9	Chr 10	Total
Raw SNPs (*n*)	7,145	5,539	4,997	4,853	5,246	3,879	3,846	4,306	3,438	3,247	46,496
Polymorphic SNPs between the parents after filtering with MAF< 5% (*n*)	1,878	1,409	1,419	1,171	1,302	1,030	1,188	1,171	842	851	12,261
Mapped SNPs in linkage map (*n*)	144	219	182	85	96	95	163	119	73	65	1,241
Average marker interval (cM)	5.61	3.14	3.86	5.84	5.77	4.15	3.49	4.12	4.97	6.23	

MAF, minor allele frequency; cM, centimorgan.

**Table 2:** Quantitative trait loci (QTLs) identified for various morphophysiological and yield-related traits under water-deficit (WD) conditions.

QTL name	Conditions	QTL type	Chromosome	Position of QTL	Left marker	Right marker	LOD	PVE (%)	Add	Interval map distance (cM)	Map distance (cM)
*qNDVI2–1*	WD	Minor	2	174	rs812099243	rs822182360	3.19	14.02	–0.02	172.5–175.5	3
*qNDVI2–2*	WD	Major	2	388	rs131350195	S2_66658066	3.93	18.59	0.03	378.5–388.5	10
*qA_net_3–1*	WD	Minor	3	314	S3_169283017	S3_173528165	2.52	14.25	–1.94	312.5–317.5	5
*qgs3–1*	WD	Minor	3	168	S3_5950551	S3_5721251	3.37	12.87	–0.02	164.5–171.5	7
*qgs6–1*	WD	Major	6	109	S6_126753475	rs836167502	4.45	18.21	–0.02	105.5–112.5	7
*qgs7–2*	WD	Minor	7	372	S7_139259301	S7_139259336	2.89	9.69	0.02	370.5–374.5	4
*qASI1–1*	WD	Minor	1	129	rs128441140	rs128842621	2.61	13.92	0.22	120.5–139.5	19
*qCH1–1*	WW, WD	Minor	1	266	S1_38965222	S1_38965211	2.52	13.84	3.53	263.5–266.5	3
*qCH1–2*	WD	Major	1	740	S1_6365045	rs818095140	3.49	15.05	4.16	738.5–741.5	3
*qCH3–1*	WD	Minor	3	235	S3_194753667	rs277236564	2.8	11.18	–3.35	232.5–239.5	7
*qCW2–1*	WW, WD	Major	2	442	S2_213060766	S2_18995588	3.03	17.95	8.59	434.5–445.5	11
*qTB2–2*	WD	Minor	2	455	S2_18995588	S2_14679066	2.58	11.5	17.91	444.5–464.5	20
*qGY2–1*	WD	Minor	2	457	S2_18995588	S2_14679066	2.78	10.54	6.2	445.5–467.5	22
*qGY6–1*	WD	Minor	6	275	S6_89546385	S6_179562549	2.54	11.33	6.41	250.5–286.5	36

cM, centimorgan; NDVI, Normalized Difference Vegetation Index; A_net_, net CO_2_ assimilation rate (μmol CO_2_ m^–2^ s^–1^); g_s_, stomatal conductance to water vapor (mol m^–2^ s^–1^); ASI, anthesis-silking interval (days); CH, cob height (cm); CW, cob weight (g/cob); TB, total biomass (g/plant); GY, grain yield (g/plant); WW, well-watered conditions; WD, water-deficit conditions; Chr, chromosome; LOD, logarithm of odds ratio; PVE (%), percentage of total phenotypic variance explained by the QTL; Add, additive effect; QTL, quantitative trait locus.

For the trait NDVI, one major and one minor QTL were detected on chr 2, with LOD scores of 3.93 and 3.19 and capturing 18.59% and 14.02% PVE, respectively. For the A_net_ trait, major and minor QTLs were detected on chr 3, with LOD scores of 3.24 and 2.52 and capturing 17.24% and 14.25% PVE, respectively. For the g_s_ trait, two major QTLs on chr 6 and three minor QTLs, one on chr 3 and two on chr 7, were detected. The LOD scores for these ranged from 2.54 to 4.45, and PVE% ranged from 9.69% to 18.21%. For the TR trait, one major QTL was detected on chr 7 (with a LOD score of 6.07 and PVE% of 21.47%), and two minor QTLs were detected, one on chr 1 (with a LOD score of 2.87 and PVE% of 10.81%) and one on chr 3 (with a LOD score of 2.72 and PVE% of 10.11%).

For the ASI trait, three minor QTLs were detected, two on chr 1 and one on chr 6, with LOD scores of 2.61, 3.02, and 3.2 and PVE% of 13.92%, 14.91%, and 13.56%, respectively. For the CH trait, one major QTL, with a LOD score of 3.49 and PVE% of 15.05%, was detected on chr 1. In addition, three minor QTLs were detected on chromosomes 1, 3, and 8, with LOD scores of 2.52, 2.8, and 2.77 and PVE% of 13.84%, 11.18%, and 14.9%, respectively. For the TB trait, three minor QTLs were detected, two on chr 2 and one on chr 5, with LOD scores of 2.53, 2.58, and 2.55 and PVE% of 13.51%, 11.50%, and 13.04%, respectively. For the GY trait, two minor QTLs were detected, one on chr 2 and one on chr 6, with LOD scores of 2.78 and 2.54 and PVE% of 10.54% and 11.33%, respectively. Finally, for the CW trait, one major QTL was detected on chr 2, with a LOD score of 3.03 and PVE% of 17.91%.

### 3.3 Identification of co-localized QTLs

Of the 27 QTLs detected under WW and WD conditions, three pairs of co-localized QTLs were identified. For traits TR and ASI, two QTLs (*qTR1–1* and *qASI1–1*) were co-localized at the marker interval rs128441140–rs128842621 on chr 1, with LOD scores of 2.87 and 2.61 and PVE% of 10.81% and 13.92%, respectively. For traits g_s_ and GY, two QTLs (*qg_s_6–2* and *qGY-6–1*) were co-localized at the marker interval S6_89546385–S6_179562549 on chr 6, with a LOD score of 2.54 and 2.54 and PVE% of 15.86% and 11.77%, respectively. Finally, for the g_s_ and TR traits, two QTLs (*qg_s_7–1* and *qTR7–1*) were co-localized at the marker interval S7_166501967–rs130671858 on chr 7, with LOD scores of 2.73 and 6.07 and PVE% of 11.3% and 21.07%, respectively.

### 3.4 Epistatic interaction among QTLs

Eleven significant digenic epistatic QTLs for traits A_net_, g_s_, TR, LT, ASI, CW, and TB were detected (**Table 3**; **Figures S2A, B**). The corresponding LOD scores ranged between 5.0 and 5.91, and PVE% ranged from 16.60% to 37.43%. The negative epistatic values (add-by-add) indicated that there was a stronger epistatic effect of recombinant genotype than of parental genotype. The epistatic effect of QTLs was negative for traits TB, A_net_, and LT, but positive for traits g_s_, ASI, TR, A_net_, and CW. The genomic region on chr 2 between the markers rs129243511 and S2_229825946 showed epistatic interaction for the CW trait on chr 8, located between the markers S8_145945904 and S8_148216407. This interaction contributed 37.43% of the PVE. Two epistatic interactions were identified for A_net_. The first involved the region on chr 1 between the markers S1_3798004 and rs128441140, which showed epistatic interaction with the region on chr 3 located between the markers rs129555629 and S3_183844216. This interaction contributed 17.60% of the PVE. For the second, a region on chr 2 between the markers rs72722896 and rs276685886 showed epistatic interaction for A_net_ with a region on chr 3, located between the markers S3_2276048 and S3_89193637. This interaction contributed 16.71% of the PVE. Finally, the genomic region on chr 2 between the markers rs131971876 and rs129196105 showed epistatic interaction for the TR trait with the region on chr 7 located between the markers S7_131791490 and S7_136261108. This interaction contributed 29.55% of the PVE.

**Table 3:** Epistatic interactions observed for morphophysiological and yield-related traits.

Trait	Condition	First chromosome	Position1 (cM)	Left marker 1	Right marker 1	Second chromosome	Position2 (cM)	Left marker 2	Right marker 2	LOD	PVE (%)	Add-by-add^a^
A_net_	WW	1	115	S1_3798004	rs128441140	3	350	rs129555629	S3_183844216	5.08	17.60	3.56
A_net_	WW	2	365	rs727228961	rs276685886	3	635	S3_2276048	S3_89193637	5.91	16.71	-3.90
g_s_	WW	6	40	S6_177038002	S6_148279548	7	120	rs130642102	rs836234066	5.32	16.60	0.05
g_s_	WW	7	270	S7_11679195	rs130488903	8	250	rs814260212	S8_73973624	5.48	18.05	0.05
TR	WD	2	265	rs131971876	rs129196105	7	365	S7_131791490	S7_136261108	5.79	29.55	0.63
LT	WD	4	60	S4_166565742	S4_182462050	6	170	rs55624911	rs130311785	5.22	34.85	-0.60
ASI	WD	2	640	S2_221752549	rs833320055	3	270	rs839843727	rs277263572	5.86	24.89	0.31
ASI	WD	1	25	S1_302248506	S1_302412690	3	210	rs131368069	rs132076316	5.00	20.18	0.27
CW	WD	2	60	rs129243511	S2_229825946	8	220	S8_145945904	S8_148216407	5.13	37.43	7.58
TB	WD	5	270	rs129997839	rs130004128	5	295	S5_24426031	S5_29004727	5.10	22. 50	-24.04
TB	WD	2	55	rs129234757	rs129243510	5	255	S5_9853336	S5_10571036	5.19	18.89	-18.16

cM, centimorgan; A_net_, net CO_2_ assimilation rate (μmol CO_2_ m^–2^ s^–1^); g_s_, stomatal conductance to water vapor (mol m^–2^ s^–1^); TR, transpiration rate (mmol H_2_O m^–2^ s^–1^); LT, leaf temperature (^°^C); ASI, anthesis-silking interval (days); CW, cob weight (g/cob); TB, total biomass (g/plant); WW, well-watered conditions; WD, water-deficit conditions; LOD, logarithm of the odds ratio, where the threshold value was ≥ 5; PVE(%), total phenotypic variance in percentage explained by the QTL; add-by-add^a^, additive-by-additive effect; negative additive effect value indicates the direction of favorable allele from donor parent; QTL, quantitative trait locus.

### 3.5 QTL–environment interaction

Using the MET (multi-environmental trials) module of ICIM, a total of 53 QTLs were identified for various traits (**Table S9**). Of these, 21 QTLs were common to those identified by the ICIM-ADD method. One QTL for each of the traits g_s_, ASI, and GY and two QTLs for the LT trait were identified using the ICIM-ADD method but were not detected by MET analysis. Conversely, the MET analysis identified 32 QTLs for traits A_net_, CH, ASI, TB, GY, NDVI, LT, and CW that had not been detected using the ICIM-ADD method. The proportion of phenotypic variation captured by additive and dominance effects [PVE(A)] ranged from 0.13% to 14.36%, and the proportion captured by additive- and dominance-by-environment effects [PVE(A by E)] for corresponding QTLs ranged from 0% to 7.92% (**Table S9**). Thus, PVE(A by E) was significantly lower than PVE(A). Most QTLs detected through the MET module of ICIM were non-significant. However, traits A_net_, ASI, g_s_, and TR were found under MET to make greater contributions to phenotypic variation (with contributions ranging from 5.24% to 21.7%), they were not found to contribute to the significant QTL × E interaction effect (**Table 4**).

**Table 4:** Quantitative trait locus (QTL) × environment (E) interactions in the recombinant inbred line (RIL) population over two seasons (rainy and post-rainy).

Trait	Chromosome	Position	Left marker	Right marker	LOD	LOD (AbyE)	PVE	PVE (AbyE)	Add	AbyE_01	AbyE_02
A_net_	3	314	S3_169283017	S3_173528165	2.55	1.1	9.51	3.55	–1.09	0.84	–0.84
A_net_	3	324	S3_174361072	rs129386882	3.23	1.35	15.53	7.92	–1.22	–1.24	1.24
ASI	1	786	rs131202973	S1_10907875	3.18	1.06	5.24	0.37	–0.09	–0.02	0.02
g_s_	3	168	S3_5950551	S3_5721251	3.33	2.17	6.14	2.64	–0.01	0.01	-0.01
g_s_	6	109	S6_126753475	rs836167502	4.66	2.15	10.17	1.81	–0.02	0.01	-0.01
g_s_	7	372	S7_139259301	S7_139259336	2.89	2.14	5.71	3.06	0.01	-0.01	0.01
g_s_	8	232	S8_152991912	S8_123369436	2.65	2.65	5.59	5.46	0.00	-0.01	0.01
TR	7	162	S7_166501967	rs130671858	6.29	1.48	21.7	7.34	0.45	0.32	–0.32

cM, centimorgan; A_net_, net CO_2_ assimilation rate (μmol CO_2_ m^–2^ s^–1^); g_s_, stomatal conductance to water vapor (mol m^–2^ s^–1^); NDVI, Normalized Difference Vegetation Index; TR, transpiration rate (mmol H_2_O m^–2^ s^–1^); LOD, logarithm of the odds ratio; LOD (AbyE), LOD score for additive- and dominance-by-environment effects; PVE (AbyE), phenotypic variation explained by additive- and dominance-by-environment effects at the current scanning position; AbyE_01, additive- and dominance-by-environment effect 1 at the current scanning position; AbyE_02, additive- and dominance-by-environment effect 2 at the current scanning position.

### 3.6 Functional annotation of SNPs

The functional annotation of genes associated with the major and minor QTLs for morphophysiological and yield-related traits and their biological/molecular functions under WD and WW conditions are shown in **Tables 5**, **S10**, respectively. The QTL regions with important genes imparting stress tolerance for different traits belonged to the categories of signal transduction (GY: *Zm00001eb297570*, protein serine/threonine phosphatase); transcription factors [NDVI: *Zm00001eb118010*, G2-like transcription factor 27; ASI: *Zm00001eb295810*, NAC-type transcription factor (NAC87)]; transporter activity (A_net_: *Zm00001eb146040*, chloride channel protein; g_s_: *Zm00001eb324180*, sugar carrier protein C; TR: *Zm00001eb015510*, phospholipid-transporting ATPase; CH: *Zm00001eb363270*, calcium-transporting ATPase); cell wall biosynthesis and its organization (TR: *Zm00001eb144960*, lipoxygenase; TR: *Zm00001eb145080*, pectin acetyl esterase); photosynthesis (g_s_ and TR: *Zm00001eb324240*, chlorophyll a/b-binding protein, chloroplast); and carbon utilization (CH: *Zm00001eb002270*, glyceraldehyde phosphate dehydrogenase B1).

**Table 5:** List of annotated genes present within quantitative trait loci (QTLs) identified under water-deficit (WD) conditions for various morphophysiological and yield traits.

QTL name	Chromosome	Position (start–end bp)	Position of SNP	SNP	Gene size (bp)	Locus ID	Annotation	Biological process
*qNDVI2–1*	2	241,929,343–241,931,437	241,930,638	G	2094	*Zm00001eb118010*	G2-like transcription factor 27 (glk27)	DNA binding
*qA_net_3–1*	3	169,275,199–169,279,402	169,283,017	C	4203	*Zm00001eb144000*	E3 ubiquitin–protein ligase RGLG1	Metal ion binding
	3	169,278,633–169,284,255	169,283,017	C	3002	*Zm00001eb144010*	Amino acid/auxin permease 20	Amino acid transport
	3	173,533,121–173,537,574	173,528,165	A	4318	*Zm00001eb145030*	BTB/POZ and TAZ domain-containing protein 3	Ubiquitin conjugation pathway
*qg_s_3–1*	3	5,718,725–5,721,785	5,721,251	G	3060	*Zm00001eb120960*	Putative transcription factor bHLH041	Protein dimerization activity
*qg_s_6–1*	6	126,751,036–126,754,755	126,753,475	A	3719	*Zm00001eb280280*	Brassinosteroid-insensitive 1-associated receptor kinase 1	Protein serine/threonine kinase activity
	6	120,726,441–120,737,326	120,736,548	G	10,885	*Zm00001eb278890*	Transcription elongation factor SPT5	mRNA binding/transcription regulation
*qg_s_7–2*	7	139,256,603–139,259,621	139,259,301	C	3018	*Zm00001eb316940*	NAD(P)-binding Rossmann-fold superfamily protein	Oxidoreductase activity
*qASI1–1*	1	53,207,544–53,208,672	53,209,688	C	1128	*Zm00001eb015510*	Phospholipid-transporting ATPase	Phospholipid translocation
*qCH1–1*	1	38,963,680–389,674,58	38,965,222	A	3565	*Zm00001eb011970*	LIM zinc-binding domain-containing protein	Cell cycle related
*qCH1–2*	1	6,360,197–6,362,989	6,365,045	A	2792	*Zm00001eb002270*	Glyceraldehyde phosphate dehydrogenase B1	Glucose metabolic process
*qCH3–1*	3	194,748,309–194,750,865	194,753,667	A	2556	*Zm00001eb151120*	Pentatricopeptide repeat-containing protein	Organellar biogenesis
	3	195,624,083–195,628,950	195,627,269	G	3257	*Zm00001eb151330*	Receptor-like serine/threonine protein kinase	Protein phosphorylation/assimilate partitioning
*qCW2–1*	2	241,494,911–241,497,812	241,498,576	G	2651	*Zm00001eb117750*	Proline-rich receptor-like protein kinase PERK4	Protein serine/threonine kinase activity
*qTB2–2*	2	14,676,667–14,679,369	14,679,066	T	2562	*Zm00001eb072580*	Osjnbb0016d16.16-like protein	–
*qGY2–1*	2	14,676,667–14,679,369	14,679,066	T	2562	*Zm00001eb072580*	Osjnbb0016d16.16-like protein	–
*qGY6–1*	6	179,555,644–179,566,050	179,562,549	C	7615	*Zm00001eb297570*	Protein serine/threonine phosphatase	Protein dephosphorylation
	6	179,563,176–179,566,051	179,562,549	C	2875	*Zm00001eb297580*	Pentatricopeptide repeat-containing protein mitochondrial	Zinc ion binding

NDVI, Normalized Difference Vegetation Index; A_net_, net CO_2_ assimilation rate (μmol CO_2_ m^–2^ s^–1^); g_s_, stomatal conductance to water vapor (mol m^–2^ s^–1^); ASI, anthesis-silking interval (days); CH, cob height (cm); CW, cob weight (g/cob); TB, total biomass (g/plant); GY, grain yield (g/plant); SNP, single nucleotide polymorphism.

### 3.7 Identification of candidate genes in the genomic region spanning QTLs under WD conditions

**Table S11** shows the names of the QTLs, their chromosome locations, left markers, right markers, QTL intervals (cM), the physical location of each QTL region (i.e., start and end), its size, and the number of genes within the QTL region. Of the 14 QTLs identified under WD conditions, two QTLs, *qg_s_7–2* and *qCH1–1*, encompassed a single gene, namely the Nicotinamide adenine dinucleotide phosphate-binding Rossmann-fold superfamily protein (*Zm00001eb316940*) and LIM homeodomain proteins transcription factor 2 (*Zm00001eb011970*), respectively. Although the physical distances between the markers of the QTLs *qg_s_6–1*, *qASI1–1*, *qCW2–1*, and *qGY6–1* were large, linkage with the trait could not be ascertained, meaning that these were not used. The physical sizes of the QTLs *qNDVI2–1*, *qNDVI2–2*, *qA_net_3–1*, *qg_s_3–1, qCH1–2*, *qCH3–1*, *qTB2–2*, and *qGY2–1* were below< 5 Mbp; these encompassed 22, 108, 264, 35, 31, 64, 372, and 372 protein-coding genes, respectively. Genes that played a significant role in WD tolerance in these QTL intervals were also identified. The details of QTLs detected in earlier studies in the QTL regions, i.e., *qA_net_3–1*, *qg_s_3–1*, *qASI1–1*, *qCH3–1*, and *qGY6–1*, are listed in **Table S12**.

## 4 Discussion

High variability was observed in various morphophysiological and yield-related traits under WW and WD conditions in a field phenotyping study of the RIL population. In particular, significant variation was observed in the NDVI, A_net_, g_s_, TR, LT, ASI, CH, CW, TB, and GY traits. The RIL population displayed transgressive segregation for traits A_net_, CH, TB, and GY under both conditions, suggesting the presence of new combinations of multiple genes with more positive or negative effects on quantitative traits than were present in either parent. The identification of progeny plants with favorable gene combinations means that they can serve as donors for further genetic improvement. The frequency distributions for the RIL subset showed near-normal distributions for most traits, indicating their polygenic nature. Positive correlations were observed between the A_net_ trait and its related traits, such as g_s_ and TR, and between yield and its related traits, such as CW, TB, and GY, under both conditions. NDVI was positively correlated with CH, CW, TB, and GY under both conditions. TR also showed a positive correlation with g_s_ as did A_net_ with g_s_ and TR, under WD conditions. Previous studies have also reported positive correlations between NDVI and GY (Trachsel et al., 2016). Interestingly, significant positive correlations were observed between morphophysiological and yield-related traits in the present study (**Table S7**), whereas only non-significant correlations were reported in an earlier study (Nikolić et al., 2012).

### 4.1 QTL mapping

The genetic map in this study consisted of 1,241 SNP markers spread across 10 linkage groups, covering a total genetic distance of 5,471.55 cM, with an average marker density of 4.4 cM per marker. RIL mapping populations are widely used for QTL identification (Li et al., 2013; Yang et al., 2016), and in this study, 27 QTLs were identified for various morphophysiological and yield-related traits under both WW conditions (13 QTLs) and WD conditions (12 QTLs). Notably, for the NDVI trait, both a major and a minor QTL were detected on chr 2, with bin position 2.10 under WD conditions. Previous studies have also reported QTLs for NDVI and plant height in two BC_1_F_2:3_ backcross populations (LPSpop and DTPpop), with 18 QTLs identified in total (Trachsel et al., 2016). In the DTPpop population, QTLs for NDVI influencing stay-green habit (*SEN6*) were also detected in bins 8.01 and 2.07. The present study identified several QTLs associated with various morphophysiological and yield-related traits under both WW and WD conditions. For the A*_net_* trait, a major QTL (under WW conditions) and a minor QTL (under WD conditions) were identified on chr 3 (bp 3.05), which also showed the presence of a minor QTL for the TR trait under WW conditions. QTLs for the TR trait were also detected on chrs 1 and 7. Two major QTLs for the g_s_ trait were identified on chr 6 (under WD conditions) and two minor QTLs, one on chr 3 (under WD conditions) and one on chr 7 (under WW conditions), were also identified. A previous study by Yu et al. (2015) identified 32 QTLs associated with chlorophyll a, chlorophyll b, total chlorophyll content, net CO_2_ photosynthetic rate, stomatal conductance, intercellular CO_2_ concentration, and TR. Another study reported on the photosynthetic performance of maize grown in drought environments (Zhao and Zhong, 2021). In a previous study conducted by Pelleschi et al. (2006), 19 major QTLs were identified for various physiological traits under drought-stressed and WW regimes. QTLs for the stomatal conductance trait have been found on all chromosomes in maize except for chr 5 (Quarrie et al., 1994; Sanguineti et al., 1999). In the present study, two minor QTLs for the LT trait (under WW conditions) were identified on chrs 6 and 8. Three minor QTLs for the ASI trait were also identified: two on chr 1 (one under WW and the other under WD conditions) and one on chr 6 (under WW conditions). A total of 33 QTLs for the ASI trait under WD stress have been reported in earlier studies, distributed across all chromosomes (Semagn et al., 2013). Additionally, QTLs for ASI under both WW and WD conditions were also identified in another study (Hu et al., 2021). In this study, we identified one major QTL under WD stress and two minor QTLs (one under WW and one under WD conditions) for cob height on chr 1, as well as one minor QTL on each of chrs 3 (under WD conditions) and 8 (under WW conditions). Previous studies have also reported QTLs for cob height on all 10 chromosomes (Beavis et al., 1994; Lubberstedt et al., 1997; Li et al., 2016), with 21 QTLs identified for both PH and CH in three common genomic regions in two biparental populations (Li et al., 2016). However, our study identified major and minor QTLs (*qCH1–2* and *qCH1–1*) at different positions on chr 1 compared with those identified in previous studies. QTLs for cob height have also been reported on chrs 3, 4, 5, 6, and 8 in previous studies, but at different bs (Yang et al., 2008; Zhao et al., 2018; Tadesse et al., 2020). Grain yield is a highly complex trait that is influenced by many genes, each exerting only a small effect (Hallauer and Miranda, 2010). Earlier studies have reported on a QTL atlas that includes the major genes for GY and its associated traits (Zhou et al., 2020). In our study, we identified QTLs for the CW and GY traits on chr 2, which were physically located at bps 241,494, 911–241,497,812 and bps 14,676,667–14,679,369, respectively. These QTLs are different from the previously reported meta-QTLs (Zhou et al., 2020). We also identified another QTL for grain yield on chr 6, located at bps 179,555,644–179,566,050, which is also different from the meta-QTLs that have been previously reported on this chromosome. Additionally, we identified three minor QTLs for the TB trait: two on chr 2 (one under WW and the other under WD conditions) and one on chr 5 (under WW conditions). Similar studies have also identified QTLs for biomass production and leaf area. For example, Chen et al. (2011) identified seven QTLs for biomass production and leaf area in the marker interval bnlg1832–P2M8/j (bp 1.05) on chr 1. Rahman et al. (2011) identified a QTL for yield on chr 1 that was co-located with the QTLs for root traits, total biomass, and osmotic potential in a region of about 15 cM. These studies suggest that genetic control of biomass and yield is complex and involves multiple QTLs distributed across different chromosomes and genomic regions.

The missing proportion of phenotypic variance for a trait may be attributed to epistasis (Carlborg and Haley, 2004), a term that refers to non-allelic interaction that can modify the degree of phenotypic expression by suppressing or enhancing the expression of interacting genes (Mackay et al., 2014; Rahman et al., 2017). Although our study was limited by a smaller population size, it is possible that epistatic effects, in addition to a few major and minor loci, contributed to the variation observed in the morphophysiological and yield-related traits studied, such as A_net_, g_s_, TR, LT, ASI, CW, and TB. However, further validation using a larger mapping population is necessary to confirm these findings. To better understand the stability of QTLs across different environments, it would be beneficial to evaluate this population in multiple locations, across multiple years, and under different treatments, such as variable temperature and watering regimes (Boer et al., 2007). Although our study used a smaller population size, a joint analysis over multiple years was conducted to establish the stability of QTLs and to evaluate the interactions between QTLs and the environment. Our findings suggest that, with the exception of g_s_ with respect to the QTL located at the marker interval S8_152991912–S8_123369436 on chr 8, most of the traits were associated with non-significant effects of environment in terms of the expression of QTLs, which captured only a small proportion of the phenotypic variation explained by additive-by-environment effects.

### 4.2 *In silico* analysis of the genomic region spanning QTLs and identification of candidate genes for stress tolerance

In this study, analysis of the genomic region of the QTLs using *in silico* methods led to the identification of candidate genes associated with stress tolerance. The QTL *qNDVI2–1* contained two important genes: Golden 2-like (GLK) transcription factor 27 (*Zm00001d007962*, *GRMZM2G173882*, and *Zm00001eb118010*) and cold-regulated 413 plasma membrane protein 2 (*Zm00001d007968*). The Golden 2-like (GLK) transcription factor 27 gene plays a crucial role in regulating chloroplast growth and development, and also contributes to the maintenance of stay-green traits (Chen et al., 2016; Qin et al., 2021), whereas the cold-regulated 413 plasma membrane protein 2 gene enhances osmotic stress tolerance through enhanced expression of *AtCor78*/*AtRD29A* (Zhou et al., 2018). In the QTL interval of *qNDVI2–2*, three genes were identified: barren stalk 2 (*Zm00001eb084940*), AP2-EREBP transcription factor 131 (*Zm00001eb084810*, *GRMZM2G087059*, and *Zm00001d003884*), and potassium high-affinity transporter 1 (*Zm00001eb084630*, *GRMZM2G093826*, and *Zm00001d00386*). These genes contribute to stress tolerance through their involvement in signaling (Yao et al., 2019), plant growth and development (Xie et al., 2019), and facilitation of potassium (K^+^) ion distribution in shoots (Qin et al., 2019). Overall, these genes in the QTL region for the NDVI trait play roles in maintaining stress tolerance through signaling and transporter activity.

In our study, we also found that the QTL interval of *qA_net_3–2* contained several genes that encode various transcription factors, such as ABI3/VP1 transcription factor 31 (*Zm00001eb144270*, *Zm00001d042460*), AP2-like ethylene-responsive transcription factor (*GRMZM2G141638*, *ZM00001EB144510*), ARR-B-transcription factor 6 (*Zm00001eb144290*, *Zm00001d042463*), bHLH transcription factor 132 (*Zm00001d042482 GRMZM2G114873*), AP2/EREBP transcription factor 53 (*Zm00001d042492*), and GRAS transcription factor 7 (*GRMZM2G013016*). These transcription factors play important roles in the regulation of downstream genes through binding to DNA elements in the promoter regions of the target genes. These genes also play vital roles in plant growth, development, hormone signaling, and stress responses (Kimotho et al., 2019). Additionally, we identified transporters, such as the magnesium (*GRMZM2G139822*, *ZM00001EB144080*) and proline transporters 1 and 2 (*ZM00001EB144010*, *GRMZM2G078024*), that are essential for maintenance of membrane homeostasis. Magnesium is a vital component of chlorophyll, which plays a crucial role in absorbance of sunlight during photosynthesis. It also acts as a phosphorus carrier in plants and is essential for phosphate metabolism (Hermans et al., 2013). Overall, our findings suggest that the *qAnet3–2* QTL region plays a vital role in the regulation of transcription factors and transporters, which are crucial for various physiological processes in plants.

The QTL *qg_s_3–1* contains three genes that are critical for plant stress tolerance and development. The bHLH transcription factor 70 (*GRMZM2G397755* and *Zm00001d039459*) is a key regulator of stress-related genes, enabling the plant to activate adaptive responses under various abiotic stresses. This transcription factor also plays a vital role in synthesis of flavonoids, which are essential for stress tolerance (Qian et al., 2021). The epidermal patterning factor-like protein (*GRMZM2G077219*, *Zm00001d039470*, and *Zm00001eb121050*) is involved in the development of stomatal cells in the upper epidermis of plant leaves (Lee et al., 2012). This protein is crucial for gaseous exchange in plants, which is vital for photosynthesis and respiration. The third gene, glutaredoxin 12 (*Grx12*) (*Zm00001eb121030*, *GRMZM2G303044*, *Zm00001d039468*, and *Zm00001eb121020*), is a stress-related redox sensor that plays a significant role in signaling through glutathione. *Grx12* enables glutathione to play a signaling role through glutathionylation of target proteins (Zaffagnini et al., 2012). The minor QTL *qg_s_7–2* includes the gene *Zm00001eb316940*, which encodes for the NAD(P)-binding Rossmann-fold superfamily protein. Although the exact function of this protein is not well understood, it is known to bind to NAD and NADP and is predicted to play a role in metabolic processes.

The QTL *qCH1–1* was expressed under both WW and WD conditions, and it is associated with the gene *Zm00001eb011970*, which encodes the LIM zinc-binding domain-containing protein DA1–2. This protein is related to ubiquitin binding and the expression of cell cycle genes, which contribute to long-distance phloem transport (Park et al., 2014). The LIM domain is a protein interaction domain that is involved in many cellular processes, including signal transduction, transcriptional regulation, and cytoskeletal organization. The QTL *qCH1–2* encompasses the gene encoding calcium-dependent protein kinase 36 (*CDPK 36*), which translates elevated calcium concentration into enhanced protein kinase activity and subsequent downstream signaling events (Singh et al., 2018). Calcium is an important secondary messenger in plants that plays a crucial role in a wide range of signaling pathways, including stress responses, development, and growth.

The QTL *qCH3–1* encompasses two important genes: receptor-like protein kinase (RLK) G-type lectin S-receptor-like serine/threonine-protein kinase (*ZM00001EB151330*), and homeobox transcription factors (*Zm00001d043231* and *ZM00001EB151130*). The first gene, RLK, is a member of the cell-surface receptor-like protein kinase family, which is critical in perception of signals. RLKs have active functions in various physiological processes such as plant growth and development, and in responses to both biotic and abiotic stresses (Ye et al., 2017). The G-type lectin S-receptor-like serine/threonine-protein kinase is involved in signaling during plant reproduction and defense against pathogens. Homeobox genes play a crucial role in specifying cell identity and positioning during embryonic development (Khan et al., 2018). They regulate various developmental processes, such as organogenesis, morphogenesis, and differentiation. The QTL *qCH3–1* includes the genes encoding RLK and homeobox-transcription factors, which play critical roles in plant growth, development, and responses to both biotic and abiotic stresses.

The QTLs *qTB2–1* and *qGY2–1* were found to be located within the same genomic region and contained several genes encoding for various transcription factors. ABI3/VP1 transcription factor 12 is involved in seed maturation and germination, and the AP2/EREBP transcription factor is known to regulate gene expression in response to various environmental cues. Calcium-dependent protein kinase 5 (*CDPK 5*) is a key component of the calcium signaling pathway and plays a crucial role in stress responses. The GRAS transcription factor is involved in various developmental processes, including root and shoot development, while MYB-related transcription factor 20 is involved in the regulation of secondary metabolism. The RING zinc finger protein-like RING/U-box superfamily protein and zinc finger C3HC4-type family of proteins are involved in protein degradation and the regulation of gene expression, respectively (Kimotho et al., 2019). In addition to these transcription factors, the QTL region also contained various stress-inducible genes, such as abscisic acid receptor *PYL9*, which plays a critical role in drought and salt stress responses, and the dehydration-responsive element-binding protein 1D, which is involved in the regulation of water stress-responsive genes. The guard cell S-type anion channel SLAC1 is involved in stomatal regulation, while the WD40 repeat-like superfamily of proteins are involved in various developmental processes, including cell division and differentiation. The naked endosperm, sucrose synthase, and xyloglucan galactosyltransferase genes are all involved in seed development and are essential for proper seed maturation.

Overall, this study has identified several candidate genes that play crucial roles in various physiological processes, including the perception of external signaling, expression of functional proteins involved in stress tolerance, and the regulation of gene expression in response to environmental cues. These findings have important implications for crop improvement, as they provide valuable insights into the molecular mechanisms underlying stress tolerance and growth and development in plants.

## 5 Conclusion

The investigation conducted in this study revealed a substantial amount of variation in a range of morphophysiological and yield-related traits in the mapping population under both WW and WD conditions. Both major and minor QTLs were identified for these traits. Interestingly, one major QTL (*qCH1–2*) and one minor QTL (*qCH1–1*) for cob height were identified at different genomic positions than in earlier studies. Co-localized QTLs were also detected for traits g_s_ and GY on chr 6 (*qg_s_6–2* and *qGY6–1*), and for traits g_s_ and TR on chr 7 (*qg_s_7–1* and *qTR7–1*). The major candidate genes associated with QTLs were also detected under WD stress conditions and were found to be involved in growth and development, senescence, ABA signaling, signal transduction, and transporter activity processes contributing to WD tolerance. To facilitate marker-assisted selection in breeding, the QTL regions identified in this study could be fine-mapped and converted into SSR markers. In addition, the putative candidate genes could be isolated and functionally characterized, and the high-yielding and better-performing RILs could be used for genetic improvement of maize.

## Data availability statement

The original contributions presented in the study are publicly available. The data can be found here: https://www.ncbi.nlm.nih.gov/sra/PRJNA913688.

## Author contributions

BS carried out the parent selection, crossing, and development of the RILs. Statistical analysis, manuscript writing, editing, and critical review of the manuscript: YV. Field phenotyping of the parents, crossing program, RIL evaluation, QTL mapping, and drafting of the manuscript: MV. Physiological characterization of the population: NRK. Assistance with data analysis: MP. Infrastructure, facilities, and manuscript editing: SKY. Field experiments and review of the manuscript: MM. Manuscript editing and review: VKS. All authors contributed to the article and approved the submitted version.
