# Supplementary material for: Mapping of QTLs for morphophysiological and yield traits under water-deficit stress and well-watered conditions in maize
Source: Front Plant Sci. 2023 May 8;14:1124619. doi: 10.3389/fpls.2023.1124619 (PMC10200936; doi:10.3389/fpls.2023.1124619)

**Mapping QTLs for morpho-physiological and yield traits under water-stressed and well-watered conditions in maize**

**B. Sarkar^*†^, Y. Varalaxmi^†^, M. Vanaja, Ravi Kumar Nakka, M. Prabhakar, S. K. Yadav, M. Maheswari, V.K. Singh**

*Division of Crop Sciences, ICAR-Central Research Institute for Dryland Agriculture, Santoshnagar, Hyderabad, Telangana, India*

**^*^Corresponding author**

**Dr. B. Sarkar**

Principal Scientist

Division of Crop Sciences

ICAR-Central Research Institute for Dryland Agriculture

Mobile : 09502311849

Email : [basudeb70@gmail.com](mailto:basudeb70@gmail.com)

**^†^ Authors contributed equally**

**Table S1: Classification of RILs (264lines) into clusters based on morphophysiological and yield related traits**

| **RIL Cluster ID** | **RIL ID number** | **Cluster distance** |
| --- | --- | --- |
| Cluster I | 39 | 36.9235 |
| Cluster I | 51 | 45.0183 |
| Cluster I | 164 | 56.0848 |
| Cluster I | 210 | 44.4114 |
| Cluster I | 33 | 36.2765 |
| Cluster I | 50 | 53.7443 |
| Cluster I | 55 | 25.4353 |
| Cluster I | 56 | 42.7211 |
| Cluster I | 69 | 32.8736 |
| Cluster I | 75 | 45.5086 |
| Cluster I | 116 | 46.6264 |
| Cluster I | 118 | 40.7669 |
| Cluster I | 128 | 33.7638 |
| Cluster I | 139 | 25.4694 |
| Cluster I | 205 | 40.2363 |
| Cluster I | 217 | 36.0686 |
| Cluster I | 228 | 28.3025 |
| Cluster I | 253 | 77.6269 |
| Cluster II | 82 | 35.4624 |
| Cluster II | 96 | 32.4818 |
| Cluster II | 104 | 30.1813 |
| Cluster II | 137 | 26.1892 |
| Cluster II | 149 | 37.621 |
| Cluster II | 197 | 38.6301 |
| Cluster II | 232 | 11.797 |
| Cluster II | 235 | 21.1437 |
| Cluster II | 250 | 34.8328 |
| Cluster II | 257 | 27.1294 |
| Cluster II | 12 | 25.0519 |
| Cluster II | 60 | 32.4292 |
| Cluster II | 64 | 28.6861 |
| Cluster II | 65 | 30.5683 |
| Cluster II | 66 | 24.7296 |
| Cluster II | 67 | 29.4499 |
| Cluster II | 80 | 29.1723 |
| Cluster II | 93 | 25.5773 |
| Cluster II | 94 | 36.4199 |
| Cluster II | 126 | 16.0285 |
| Cluster II | 129 | 29.521 |
| Cluster II | 141 | 31.5011 |
| Cluster II | 146 | 18.9754 |
| Cluster II | 147 | 26.4455 |
| Cluster II | 155 | 36.1051 |
| Cluster II | 160 | 24.6625 |
| Cluster II | 163 | 32.2013 |
| Cluster II | 171 | 19.9486 |
| Cluster II | 203 | 29.3868 |
| Cluster II | 209 | 22.518 |
| Cluster II | 215 | 34.5296 |
| Cluster II | 220 | 26.1961 |
| Cluster II | 234 | 20.6164 |
| Cluster II | 236 | 18.8018 |
| Cluster II | 247 | 28.2254 |
| Cluster II | 249 | 35.4765 |
| Cluster II | 258 | 19.415 |
| Cluster II | 260 | 18.0916 |
| Cluster III | 2 | 31.6056 |
| Cluster III | 16 | 29.526 |
| Cluster III | 22 | 21.6629 |
| Cluster III | 91 | 41.6618 |
| Cluster III | 100 | 38.0016 |
| Cluster III | 173 | 28.9967 |
| Cluster III | 179 | 19.7415 |
| Cluster III | 181 | 35.2722 |
| Cluster III | 189 | 35.989 |
| Cluster III | 261 | 32.7708 |
| Cluster III | 263 | 56.8034 |
| Cluster III | 3 | 27.565 |
| Cluster III | 11 | 17.2039 |
| Cluster III | 21 | 42.7597 |
| Cluster III | 70 | 33.6027 |
| Cluster III | 73 | 25.0082 |
| Cluster III | 76 | 45.4546 |
| Cluster III | 77 | 17.9131 |
| Cluster III | 83 | 46.5769 |
| Cluster III | 85 | 34.1581 |
| Cluster III | 92 | 40.5298 |
| Cluster III | 105 | 20.3383 |
| Cluster III | 133 | 26.5695 |
| Cluster III | 162 | 26.8102 |
| Cluster III | 180 | 40.6403 |
| Cluster III | 194 | 31.725 |
| Cluster IV | 36 | 31.7181 |
| Cluster IV | 48 | 42.5831 |
| Cluster IV | 49 | 22.0932 |
| Cluster IV | 97 | 35.9411 |
| Cluster IV | 136 | 19.1839 |
| Cluster IV | 138 | 26.3538 |
| Cluster IV | 148 | 30.0551 |
| Cluster IV | 225 | 36.5581 |
| Cluster IV | 237 | 23.4826 |
| Cluster IV | 5 | 37.802 |
| Cluster IV | 8 | 39.5778 |
| Cluster IV | 31 | 35.2695 |
| Cluster IV | 32 | 47.2448 |
| Cluster IV | 37 | 19.5713 |
| Cluster IV | 41 | 33.9018 |
| Cluster IV | 42 | 37.6678 |
| Cluster IV | 47 | 32.2989 |
| Cluster IV | 54 | 26.5977 |
| Cluster IV | 59 | 31.7091 |
| Cluster IV | 71 | 45.8197 |
| Cluster IV | 101 | 26.3553 |
| Cluster IV | 109 | 36.7498 |
| Cluster IV | 113 | 20.8445 |
| Cluster IV | 120 | 33.9199 |
| Cluster IV | 121 | 32.1869 |
| Cluster IV | 122 | 28.5767 |
| Cluster IV | 125 | 43.3295 |
| Cluster IV | 130 | 18.9782 |
| Cluster IV | 140 | 33.9096 |
| Cluster IV | 145 | 30.5353 |
| Cluster IV | 158 | 30.8875 |
| Cluster IV | 169 | 27.312 |
| Cluster IV | 183 | 34.9116 |
| Cluster IV | 186 | 42.895 |
| Cluster IV | 190 | 26.8046 |
| Cluster IV | 207 | 24.0318 |
| Cluster IV | 211 | 21.7572 |
| Cluster IV | 213 | 33.7894 |
| Cluster IV | 214 | 25.3754 |
| Cluster IV | 226 | 25.4232 |
| Cluster IV | 227 | 30.3703 |
| Cluster IV | 229 | 42.7122 |
| Cluster V | 87 | 21.8937 |
| Cluster V | 106 | 29.0218 |
| Cluster V | 110 | 25.0653 |
| Cluster V | 176 | 19.9349 |
| Cluster V | 246 | 34.9058 |
| Cluster V | 248 | 26.5936 |
| Cluster V | 251 | 51.4387 |
| Cluster V | 255 | 31.9817 |
| Cluster V | 259 | 32.2222 |
| Cluster V | 265 | 13.4058 |
| Cluster V | 1 | 35.6158 |
| Cluster V | 6 | 26.1426 |
| Cluster V | 44 | 25.3103 |
| Cluster V | 84 | 21.2394 |
| Cluster V | 88 | 32.592 |
| Cluster V | 89 | 20.5242 |
| Cluster V | 90 | 29.4134 |
| Cluster V | 111 | 38.4317 |
| Cluster V | 112 | 23.2751 |
| Cluster V | 114 | 25.8229 |
| Cluster V | 154 | 45.6747 |
| Cluster V | 159 | 26.551 |
| Cluster V | 166 | 33.3787 |
| Cluster V | 170 | 36.4954 |
| Cluster V | 172 | 29.0387 |
| Cluster V | 174 | 19.9779 |
| Cluster V | 175 | 20.8022 |
| Cluster V | 178 | 15.3836 |
| Cluster V | 184 | 24.0386 |
| Cluster V | 188 | 34.5679 |
| Cluster V | 195 | 23.7561 |
| Cluster V | 199 | 27.6579 |
| Cluster V | 200 | 26.6814 |
| Cluster V | 202 | 38.8485 |
| Cluster V | 206 | 34.2314 |
| Cluster V | 216 | 29.0431 |
| Cluster V | 218 | 25.2956 |
| Cluster V | 222 | 22.5357 |
| Cluster V | 241 | 27.9264 |
| Cluster V | 256 | 29.9704 |
| Cluster V | 264 | 35.2616 |
| Cluster V | 266 | 38.4012 |
| Cluster V | 267 | 14.0185 |
| Cluster VI | 10 | 24.7223 |
| Cluster VI | 14 | 52.6681 |
| Cluster VI | 28 | 26.732 |
| Cluster VI | 52 | 33.9335 |
| Cluster VI | 57 | 29.2689 |
| Cluster VI | 78 | 31.922 |
| Cluster VI | 123 | 30.2894 |
| Cluster VI | 131 | 34.7562 |
| Cluster VI | 187 | 23.3027 |
| Cluster VI | 192 | 30.7476 |
| Cluster VI | 193 | 17.5791 |
| Cluster VI | 204 | 32.2824 |
| Cluster VI | 208 | 12.8511 |
| Cluster VI | 231 | 26.3575 |
| Cluster VI | 243 | 35.6199 |
| Cluster VI | 9 | 25.2377 |
| Cluster VI | 13 | 35.9218 |
| Cluster VI | 15 | 30.823 |
| Cluster VI | 18 | 39.1623 |
| Cluster VI | 20 | 36.5582 |
| Cluster VI | 25 | 29.4955 |
| Cluster VI | 26 | 38.5037 |
| Cluster VI | 29 | 37.5089 |
| Cluster VI | 30 | 28.0956 |
| Cluster VI | 34 | 26.3694 |
| Cluster VI | 40 | 20.8996 |
| Cluster VI | 46 | 20.4721 |
| Cluster VI | 53 | 35.6984 |
| Cluster VI | 98 | 24.8265 |
| Cluster VI | 99 | 28.7652 |
| Cluster VI | 102 | 36.1564 |
| Cluster VI | 103 | 48.912 |
| Cluster VI | 107 | 24.917 |
| Cluster VI | 124 | 29.2631 |
| Cluster VI | 132 | 20.8511 |
| Cluster VI | 150 | 22.6901 |
| Cluster VI | 156 | 42.2958 |
| Cluster VI | 191 | 29.054 |
| Cluster VI | 196 | 30.1896 |
| Cluster VI | 198 | 41.8079 |
| Cluster VI | 212 | 13.5296 |
| Cluster VI | 221 | 21.3794 |
| Cluster VI | 230 | 33.7998 |
| Cluster VI | 245 | 36.5567 |
| Cluster VII | 17 | 35.2617 |
| Cluster VII | 19 | 37.9174 |
| Cluster VII | 23 | 56.2859 |
| Cluster VII | 35 | 18.4288 |
| Cluster VII | 43 | 40.8181 |
| Cluster VII | 61 | 27.0474 |
| Cluster VII | 79 | 15.3546 |
| Cluster VII | 81 | 28.2092 |
| Cluster VII | 115 | 43.8974 |
| Cluster VII | 134 | 23.3982 |
| Cluster VII | 157 | 32.3208 |
| Cluster VII | 161 | 20.8904 |
| Cluster VII | 168 | 28.8216 |
| Cluster VII | 223 | 25.8747 |
| Cluster VII | 244 | 39.2584 |
| Cluster VII | 4 | 22.7879 |
| Cluster VII | 7 | 31.7786 |
| Cluster VII | 24 | 24.5593 |
| Cluster VII | 38 | 38.0563 |
| Cluster VII | 45 | 23.9399 |
| Cluster VII | 58 | 17.6415 |
| Cluster VII | 62 | 27.1097 |
| Cluster VII | 63 | 33.8909 |
| Cluster VII | 68 | 19.5016 |
| Cluster VII | 72 | 19.1231 |
| Cluster VII | 74 | 24.9251 |
| Cluster VII | 86 | 21.662 |
| Cluster VII | 95 | 31.0496 |
| Cluster VII | 108 | 26.0288 |
| Cluster VII | 117 | 20.7973 |
| Cluster VII | 119 | 39.457 |
| Cluster VII | 127 | 25.7737 |
| Cluster VII | 135 | 30.0107 |
| Cluster VII | 142 | 27.3909 |
| Cluster VII | 143 | 16.7079 |
| Cluster VII | 151 | 28.1868 |
| Cluster VII | 152 | 29.2139 |
| Cluster VII | 153 | 35.7319 |
| Cluster VII | 167 | 27.3369 |
| Cluster VII | 177 | 44.0029 |
| Cluster VII | 182 | 19.3628 |
| Cluster VII | 185 | 18.9121 |
| Cluster VII | 201 | 25.7352 |
| Cluster VII | 219 | 21.7684 |
| Cluster VII | 224 | 44.823 |
| Cluster VII | 238 | 27.6499 |
| Cluster VII | 239 | 26.8691 |
| Cluster VII | 240 | 19.3451 |
| Cluster VII | 242 | 32.4219 |
| Cluster VII | 262 | 31.7706 |
| Cluster VIII | 144 | 41.0227 |
| Cluster VIII | 233 | 25.5859 |
| Cluster VIII | 27 | 39.8134 |

**Table S2 A sub-set of genotypes selected from each cluster for SNP genotyping**

| **RIL cluster** | **No. of RILs in the cluster** | **No. of RILs selected for SNP genotyping** | **IDs of selected RILs** |
| --- | --- | --- | --- |
| Cluster I | 18 | 4 | RIL39, RIL51, RIL164, RIL210 |
| Cluster II | 38 | 9 | RIL82, RIL137, RIL146, RIL149, RIL163, RIL197, RIL236, RIL250, RIL257 |
| Cluster III | 26 | 10 | RIL16, RIL22, RIL83, RIL91, RIL173, RIL179, RIL181, RIL189, RIL261, RIL263 |
| Cluster IV | 42 | 12 | RIL48, RIL49, RIL97, RIL120, RIL130, RIL136, RIL138, RIL148, RIL158, RIL225,  RIL226, RIL237 |
| Cluster V | 43 | 13 | RIL1, RIL87, RIL106, RIL110, RIL176, RIL199, RIL200, RIL206, RIL246, RIL248  RIL251, RIL255, RIL265 |
| Cluster VI | 44 | 12 | RIL10, RIL14, RIL52, RIL57, RIL78, RIL187, RIL192, RIL193, RIL204, RIL208  RIL231, RIL243 |
| Cluster VII | 50 | 17 | RIL17, RIL19, RIL23, RIL35, RIL43, RIL62, RIL79, RIL81, RIL115, RIL134, RIL157, RIL161, RIL223, RIL224, RIL240  RIL242, RIL244 |
| Cluster VIII | 3 | 2 | RIL144, RIL233 |
| **Total** | **264** | **79** |  |

**Table S3 Two seasons mean of morpho- physiological traits of parents and recombinant inbred lines (RILs)**

| Genotype | NDVI_  WW | NDVI_  WD | A_net__  WW | A_net__  WD | g_s__  WW | g_s__  WD | TR_  WW | TR_  WD | LT_  WW | LT_  WD | ASI_  WW | ASI_  WD | CH_  WW | CH_  WD | CW_  WW | CW_  WD | GY_  WW | GY_  WD | TB_  WW | TB_  WD |
| --- | --- | --- | --- | --- | --- | --- | --- | --- | --- | --- | --- | --- | --- | --- | --- | --- | --- | --- | --- | --- |
| HKI161 | 0.73 | 0.62 | 37.28 | 12.69 | 0.36 | 0.29 | 11.87 | 7.0 | 37.18 | 36.55 | 3 | 2 | 66.5 | 53.17 | 80.33 | 62 | 49 | 33.67 | 134.5 | 114.17 |
| SNJ201126 | 0.77 | 0.65 | 43.33 | 16.73 | 0.4 | 0.33 | 13.93 | 8.67 | 30.78 | 32.32 | 2 | 3 | 81.67 | 61.83 | 89.83 | 80 | 66 | 40.83 | 140.83 | 121.17 |
| RIL1 | 0.82 | 0.76 | 37.52 | 15.62 | 0.3 | 0.14 | 6.23 | 4.11 | 28.62 | 31.33 | 3 | 3 | 88.33 | 82.83 | 68.41 | 57.05 | 49.51 | 35.28 | 247.89 | 154.13 |
| RIL2 | 0.82 | 0.71 | 33.32 | 20.98 | 0.43 | 0.22 | 9.92 | 5.8 | 30.86 | 30.7 | 3 | 3 | 82.50 | 67.00 | 118.69 | 102.82 | 94.94 | 52.95 | 216.29 | 159.51 |
| RIL3 | 0.76 | 0.77 | 50.4 | 10.29 | 0.45 | 0.14 | 11.24 | 4.05 | 29.7 | 32.15 | 3 | 3 | 80.67 | 63.33 | 90.53 | 76.26 | 65.64 | 51.93 | 212.79 | 155.81 |
| RIL4 | 0.82 | 0.53 | 38.52 | 15.52 | 0.29 | 0.19 | 7.8 | 4.79 | 30.2 | 31.34 | 3 | 3 | 73.50 | 60.00 | 90.77 | 73.61 | 64.02 | 48.07 | 207.34 | 138.37 |
| RIL5 | 0.74 | 0.63 | 42.82 | 22.5 | 0.29 | 0.25 | 6.63 | 6.5 | 29.65 | 31.1 | 3 | 3 | 79.50 | 76.67 | 101.64 | 87.79 | 79.48 | 62.37 | 206.58 | 183.90 |
| RIL6 | 0.75 | 0.63 | 48.58 | 21.3 | 0.39 | 0.24 | 8.06 | 5.81 | 28.9 | 30.08 | 3 | 3 | 78.50 | 65.17 | 102.62 | 90.05 | 70.96 | 53.26 | 199.12 | 187.06 |
| RIL7 | 0.8 | 0.73 | 26.82 | 9.17 | 0.22 | 0.16 | 5.54 | 4.25 | 30.62 | 30.94 | 2 | 2 | 96.67 | 92.50 | 137.17 | 110.20 | 100.35 | 64.28 | 310.12 | 238.39 |
| RIL8 | 0.76 | 0.67 | 38.83 | 12.05 | 0.39 | 0.25 | 9.04 | 5.46 | 29.14 | 31.32 | 3 | 3 | 65.83 | 53.67 | 96.45 | 80.66 | 61.26 | 51.21 | 214.16 | 216.38 |
| RIL9 | 0.74 | 0.65 | 33.42 | 25.35 | 0.32 | 0.35 | 6.94 | 9.33 | 30.43 | 31.61 | 3 | 3 | 74.83 | 61.83 | 132.52 | 73.51 | 84.52 | 47.94 | 270.80 | 151.40 |
| RIL10 | 0.74 | 0.74 | 27.72 | 23.42 | 0.22 | 0.21 | 6.69 | 5.7 | 30.33 | 34.03 | 3 | 3 | 76.83 | 61.00 | 98.53 | 63.82 | 64.83 | 42.89 | 204.91 | 160.01 |
| RIL11 | 0.71 | 0.65 | 38.83 | 12.98 | 0.34 | 0.13 | 8.72 | 3.74 | 30.09 | 32.09 | 2 | 2 | 91.33 | 55.17 | 91.31 | 74.83 | 70.34 | 39.13 | 259.94 | 150.87 |
| RIL12 | 0.76 | 0.61 | 42.23 | 22.22 | 0.37 | 0.18 | 7.28 | 4.98 | 28.65 | 30.75 | 3 | 3 | 106.00 | 85.33 | 83.48 | 67.22 | 62.88 | 48.49 | 236.20 | 177.63 |
| RIL13 | 0.78 | 0.75 | 47.25 | 20.1 | 0.44 | 0.2 | 9.58 | 5.4 | 30.18 | 31.31 | 3 | 3 | 83.00 | 68.00 | 102.38 | 87.14 | 83.25 | 62.48 | 220.05 | 206.08 |
| RIL14 | 0.78 | 0.75 | 45.17 | 9.58 | 0.48 | 0.12 | 11.33 | 3.36 | 29.61 | 31.82 | 3 | 2 | 90.33 | 76.67 | 115.34 | 95.29 | 86.98 | 57.03 | 239.34 | 200.21 |
| RIL15 | 0.79 | 0.54 | 42.53 | 24.2 | 0.26 | 0.16 | 5.63 | 4.53 | 29.12 | 30.51 | 3 | 3 | 93.33 | 64.67 | 68.98 | 58.25 | 52.39 | 36.75 | 146.53 | 133.71 |
| RIL16 | 0.8 | 0.58 | 38.65 | 16.42 | 0.34 | 0.15 | 7.03 | 4.1 | 29.08 | 31.04 | 3 | 2 | 76.83 | 63.83 | 76.65 | 64.81 | 56.87 | 52.27 | 178.34 | 144.60 |
| RIL17 | 0.7 | 0.63 | 28.32 | 15.75 | 0.24 | 0.19 | 5.67 | 5.11 | 29.08 | 31.43 | 3 | 2 | 67.83 | 47.17 | 77.38 | 65.27 | 56.62 | 49.34 | 137.38 | 137.96 |
| RIL18 | 0.79 | 0.64 | 43.22 | 25 | 0.32 | 0.18 | 8 | 5.12 | 30.89 | 31.62 | 3 | 2 | 105.83 | 71.50 | 116.64 | 97.77 | 88.72 | 59.70 | 329.20 | 200.88 |
| RIL19 | 0.78 | 0.68 | 42.93 | 17.92 | 0.29 | 0.18 | 8.17 | 4.45 | 29.38 | 31.56 | 2 | 3 | 82.17 | 69.50 | 77.03 | 62.67 | 66.29 | 43.88 | 220.73 | 159.33 |
| RIL20 | 0.76 | 0.72 | 42.73 | 19.53 | 0.33 | 0.19 | 8.24 | 4.6 | 29.08 | 31.56 | 3 | 2 | 75.33 | 71.17 | 91.57 | 73.96 | 69.14 | 47.40 | 237.23 | 180.15 |
| RIL21 | 0.77 | 0.68 | 34.58 | 15.48 | 0.28 | 0.17 | 7 | 4.55 | 29.05 | 31.61 | 3 | 3 | 96.67 | 80.17 | 64.67 | 55.58 | 47.58 | 34.38 | 208.72 | 153.38 |
| RIL22 | 0.83 | 0.73 | 27.25 | 14.39 | 0.32 | 0.17 | 7.21 | 4.63 | 29.27 | 31.89 | 3 | 3 | 94.33 | 84.00 | 95.14 | 80.48 | 69.78 | 49.81 | 213.50 | 168.95 |
| RIL23 | 0.75 | 0.69 | 35.22 | 12.36 | 0.33 | 0.11 | 8.45 | 2.17 | 30.52 | 32.49 | 2 | 3 | 77.00 | 69.83 | 66.03 | 50.85 | 43.72 | 21.81 | 204.92 | 121.82 |
| RIL24 | 0.77 | 0.71 | 33.92 | 13.08 | 0.33 | 0.15 | 8.52 | 4.22 | 30.5 | 32.31 | 3 | 3 | 85.83 | 63.83 | 51.66 | 44.50 | 40.37 | 39.34 | 164.90 | 115.72 |
| RIL25 | 0.77 | 0.62 | 40.68 | 17.6 | 0.44 | 0.24 | 9.83 | 6.02 | 30.47 | 31.13 | 3 | 3 | 81.50 | 72.33 | 122.63 | 100.20 | 91.08 | 52.50 | 267.65 | 209.39 |
| RIL26 | 0.74 | 0.67 | 29.35 | 16.97 | 0.31 | 0.18 | 7.3 | 5.17 | 30.77 | 31.55 | 2 | 3 | 72.33 | 63.83 | 106.98 | 85.49 | 77.07 | 67.42 | 235.12 | 246.57 |
| RIL27 | 0.79 | 0.71 | 35.4 | 15.27 | 0.23 | 0.16 | 5.39 | 4.33 | 29.28 | 30.41 | 3 | 3 | 85.33 | 78.50 | 93.19 | 79.20 | 78.44 | 41.21 | 254.42 | 172.17 |
| RIL28 | 0.73 | 0.65 | 34.27 | 16.59 | 0.28 | 0.15 | 7.68 | 4.2 | 29.89 | 32.34 | 3 | 3 | 82.00 | 72.00 | 94.49 | 64.11 | 66.28 | 36.25 | 177.36 | 143.06 |

| Genotype | NDVI_  WW | NDVI_  WD | AN_  WW | AN_  WD | g_s__  WW | g_s__  WD | TR_  WW | TR_  WD | LT_  WW | LT_  WD | ASI_  WW | ASI_  WD | CH_  WW | CH_  WD | CW_  WW | CW_  WD | GY_  WW | GY_  WD | TB_  WW | TB_  WD |
| --- | --- | --- | --- | --- | --- | --- | --- | --- | --- | --- | --- | --- | --- | --- | --- | --- | --- | --- | --- | --- |
| RIL29 | 0.75 | 0.64 | 41.43 | 25.6 | 0.42 | 0.23 | 9.19 | 6.32 | 30.26 | 31.49 | 2 | 3 | 82.33 | 73.17 | 108.46 | 73.25 | 73.87 | 43.64 | 216.49 | 176.55 |
| RIL30 | 0.8 | 0.76 | 43.92 | 28.38 | 0.45 | 0.18 | 10.46 | 5.09 | 30.51 | 31.5 | 3 | 3 | 86.00 | 73.33 | 128.96 | 88.11 | 83.96 | 59.67 | 262.54 | 219.58 |
| RIL31 | 0.83 | 0.75 | 36.43 | 23.4 | 0.31 | 0.26 | 7.48 | 6.66 | 30.2 | 30.66 | 2 | 2 | 88.17 | 87.67 | 103.74 | 93.04 | 86.57 | 75.61 | 289.51 | 211.54 |
| RIL32 | 0.8 | 0.66 | 32.3 | 20.78 | 0.46 | 0.22 | 8.73 | 6.63 | 29.22 | 31.85 | 2 | 3 | 68.33 | 66.67 | 118.68 | 81.31 | 94.92 | 34.90 | 262.05 | 191.56 |
| RIL33 | 0.77 | 0.72 | 40.28 | 17.41 | 0.29 | 0.12 | 7.29 | 4.06 | 29.92 | 33.32 | 3 | 3 | 102.67 | 85.00 | 107.51 | 69.40 | 65.67 | 48.09 | 206.16 | 202.40 |
| RIL34 | 0.73 | 0.62 | 32.65 | 12.95 | 0.29 | 0.15 | 6.3 | 3.6 | 28.77 | 30.05 | 3 | 3 | 82.00 | 58.33 | 107.39 | 75.79 | 85.74 | 52.45 | 226.12 | 163.68 |
| RIL35 | 0.76 | 0.7 | 33.67 | 14.25 | 0.31 | 0.14 | 7.71 | 3.95 | 30.54 | 32.25 | 3 | 3 | 80.00 | 66.17 | 93.20 | 64.60 | 52.96 | 38.77 | 203.47 | 155.50 |
| RIL36 | 0.77 | 0.72 | 38.25 | 17.11 | 0.31 | 0.21 | 7.99 | 5.14 | 29.44 | 31.22 | 2 | 3 | 88.83 | 66.33 | 116.61 | 95.09 | 92.53 | 42.17 | 292.50 | 125.70 |
| RIL37 | 0.72 | 0.64 | 38.23 | 24.27 | 0.34 | 0.21 | 6.6 | 6.14 | 28.59 | 31.46 | 3 | 3 | 71.50 | 64.83 | 77.14 | 64.11 | 56.38 | 42.64 | 188.80 | 152.16 |
| RIL38 | 0.75 | 0.66 | 39.4 | 12.58 | 0.37 | 0.15 | 7.29 | 3.69 | 29.53 | 29.77 | 2 | 3 | 76.67 | 69.83 | 84.99 | 77.30 | 71.45 | 42.66 | 209.04 | 148.06 |
| RIL39 | 0.77 | 0.69 | 35.08 | 12.43 | 0.35 | 0.17 | 8.14 | 4.72 | 30.09 | 32.09 | 3 | 3 | 78.00 | 63.50 | 94.50 | 64.07 | 67.11 | 39.43 | 235.66 | 147.73 |
| RIL40 | 0.71 | 0.62 | 44.42 | 18.02 | 0.32 | 0.18 | 7.34 | 5.15 | 30.23 | 31.14 | 3 | 2 | 77.50 | 74.50 | 71.63 | 65.84 | 56.04 | 34.14 | 203.59 | 140.78 |
| RIL41 | 0.73 | 0.64 | 41.42 | 18.27 | 0.41 | 0.21 | 9.77 | 5.55 | 30.2 | 32.17 | 3 | 3 | 64.33 | 57.33 | 96.06 | 71.85 | 65.29 | 36.76 | 218.12 | 176.40 |
| RIL42 | 0.78 | 0.74 | 38.95 | 20.88 | 0.36 | 0.22 | 8.67 | 6.05 | 29.94 | 31.98 | 2 | 3 | 83.67 | 82.67 | 94.02 | 79.62 | 63.68 | 53.55 | 233.53 | 177.67 |
| RIL43 | 0.73 | 0.59 | 40.6 | 9.00 | 0.39 | 0.15 | 9.82 | 3.69 | 30.77 | 32.15 | 3 | 2 | 87.00 | 58.83 | 91.42 | 66.44 | 71.25 | 46.08 | 176.77 | 137.98 |
| RIL44 | 0.76 | 0.65 | 43.25 | 23.63 | 0.34 | 0.2 | 6.9 | 5.1 | 28.82 | 30.02 | 3 | 3 | 101.83 | 77.67 | 70.56 | 61.08 | 56.90 | 38.39 | 159.31 | 168.91 |
| RIL45 | 0.69 | 0.56 | 37.72 | 16.85 | 0.35 | 0.15 | 7.37 | 4.03 | 28.97 | 30.46 | 2 | 2 | 76.50 | 64.17 | 89.09 | 81.55 | 60.71 | 50.20 | 268.95 | 194.57 |
| RIL46 | 0.77 | 0.67 | 43.57 | 24.78 | 0.43 | 0.42 | 9.93 | 9.01 | 28.92 | 30.98 | 3 | 3 | 78.50 | 69.00 | 101.98 | 92.32 | 71.43 | 47.78 | 172.19 | 137.45 |
| RIL47 | 0.69 | 0.56 | 45.97 | 16.28 | 0.31 | 0.19 | 6.93 | 4.63 | 29.17 | 30.1 | 4 | 3 | 77.67 | 68.67 | 108.80 | 91.71 | 75.83 | 42.17 | 233.74 | 141.40 |
| RIL48 | 0.68 | 0.66 | 29.98 | 14.82 | 0.22 | 0.16 | 4.56 | 4.38 | 28.62 | 30.22 | 3 | 3 | 74.00 | 65.83 | 79.61 | 64.40 | 56.87 | 35.39 | 201.23 | 126.99 |
| RIL49 | 0.74 | 0.67 | 34.48 | 14.00 | 0.27 | 0.14 | 6.58 | 3.69 | 29.17 | 31.91 | 3 | 2 | 96.33 | 89.83 | 131.58 | 108.19 | 102.12 | 55.18 | 271.65 | 163.26 |
| RIL50 | 0.76 | 0.66 | 31.72 | 14.27 | 0.22 | 0.15 | 5.46 | 4.1 | 29.36 | 32.0 | 3 | 3 | 79.33 | 59.67 | 99.31 | 80.55 | 73.05 | 36.50 | 222.17 | 119.17 |
| RIL51 | 0.79 | 0.68 | 27.02 | 19.87 | 0.46 | 0.24 | 10.08 | 5.67 | 29.37 | 31.97 | 3 | 3 | 95.67 | 75.33 | 89.31 | 78.52 | 69.63 | 39.04 | 198.00 | 146.58 |
| RIL52 | 0.76 | 0.76 | 36.23 | 22.43 | 0.31 | 0.2 | 7.98 | 4.85 | 29.74 | 31.46 | 2 | 3 | 92.83 | 71.17 | 109.57 | 82.65 | 86.26 | 62.83 | 216.31 | 180.34 |
| RIL53 | 0.75 | 0.71 | 31.00 | 18.02 | 0.28 | 0.24 | 7.02 | 5.84 | 29.97 | 31.32 | 3 | 3 | 69.50 | 63.00 | 94.70 | 81.66 | 76.27 | 62.38 | 197.09 | 153.24 |
| RIL54 | 0.71 | 0.64 | 34.18 | 18.64 | 0.28 | 0.21 | 7.4 | 5.98 | 29.78 | 32.17 | 3 | 4 | 79.83 | 53.83 | 88.63 | 70.47 | 67.60 | 61.37 | 240.23 | 165.73 |
| RIL55 | 0.8 | 0.76 | 31.18 | 19.45 | 0.27 | 0.18 | 6.68 | 4.58 | 29.94 | 31.94 | 3 | 4 | 88.17 | 81.33 | 65.19 | 67.05 | 34.79 | 45.80 | 208.10 | 197.45 |
| RIL56 | 0.8 | 0.7 | 46.03 | 19.88 | 0.36 | 0.28 | 7.09 | 6.55 | 28.46 | 30.17 | 3 | 3 | 90.17 | 63.67 | 85.70 | 67.62 | 67.91 | 51.20 | 196.61 | 159.54 |
| RIL57 | 0.78 | 0.63 | 36.82 | 26.97 | 0.3 | 0.22 | 6.61 | 6.05 | 29.78 | 31.25 | 2 | 2 | 101.17 | 77.00 | 80.81 | 63.87 | 60.75 | 43.99 | 192.46 | 145.90 |
| RIL58 | 0.72 | 0.72 | 24.5 | 17.13 | 0.23 | 0.18 | 5.42 | 4.52 | 30.65 | 30.77 | 3 | 2 | 78.33 | 67.67 | 61.40 | 45.38 | 42.43 | 31.65 | 186.53 | 158.54 |
| RIL59 | 0.74 | 0.66 | 35.52 | 14.6 | 0.42 | 0.23 | 9.37 | 6.35 | 30.55 | 31.52 | 3 | 3 | 80.50 | 75.33 | 130.73 | 98.02 | 89.28 | 63.83 | 243.30 | 219.30 |

| Genotype | NDVI_  WW | NDVI_  WD | AN_  WW | AN_  WD | g_s__  WW | g_s__  WD | TR_  WW | TR_  WD | LT_  WW | LT_  WD | ASI_  WW | ASI_  WD | CH_  WW | CH_  WD | CW_  WW | CW_  WD | GY_  WW | GY_  WD | TB_  WW | TB_  WD |
| --- | --- | --- | --- | --- | --- | --- | --- | --- | --- | --- | --- | --- | --- | --- | --- | --- | --- | --- | --- | --- |
| RIL60 | 0.78 | 0.72 | 39.43 | 13.83 | 0.4 | 0.14 | 9.01 | 3.18 | 29.63 | 31.85 | 3 | 4 | 102.33 | 75.17 | 103.71 | 83.83 | 81.96 | 48.27 | 249.10 | 184.89 |
| RIL61 | 0.74 | 0.61 | 39.45 | 21.45 | 0.31 | 0.22 | 6.19 | 5.75 | 28.52 | 30.54 | 2 | 2 | 91.67 | 71.67 | 73.47 | 61.21 | 53.26 | 29.44 | 175.34 | 170.19 |
| RIL62 | 0.81 | 0.75 | 44 | 21.37 | 0.47 | 0.22 | 10.59 | 5.86 | 30.28 | 30.71 | 3 | 3 | 87.33 | 79.50 | 121.97 | 93.91 | 84.99 | 75.40 | 239.52 | 228.80 |
| RIL63 | 0.68 | 0.5 | 36.28 | 23.83 | 0.19 | 0.24 | 4.87 | 5.24 | 28.99 | 30.66 | 3 | 3 | 88.33 | 59.83 | 60.84 | 48.58 | 46.20 | 35.79 | 175.26 | 124.79 |
| RIL64 | 0.66 | 0.49 | 35.95 | 24.6 | 0.3 | 0.24 | 6.24 | 6.4 | 29.36 | 30.89 | 3 | 3 | 89.50 | 61.67 | 66.11 | 51.80 | 48.77 | 29.03 | 179.66 | 128.92 |
| RIL65 | 0.78 | 0.71 | 31.25 | 26.27 | 0.2 | 0.22 | 5.92 | 6.21 | 29.86 | 31.98 | 3 | 2 | 94.67 | 63.17 | 110.20 | 74.12 | 71.88 | 38.57 | 281.87 | 161.57 |
| RIL66 | 0.83 | 0.6 | 38.25 | 31.23 | 0.29 | 0.28 | 6.68 | 6.91 | 28.83 | 30.66 | 3 | 4 | 85.00 | 72.33 | 110.69 | 69.57 | 63.72 | 53.83 | 203.28 | 164.45 |
| RIL67 | 0.8 | 0.67 | 38.4 | 17.17 | 0.35 | 0.18 | 7.33 | 4.31 | 30.29 | 30.17 | 2 | 3 | 88.67 | 81.50 | 101.13 | 57.36 | 60.01 | 37.23 | 198.65 | 147.02 |
| RIL68 | 0.76 | 0.67 | 35.33 | 11.19 | 0.28 | 0.13 | 7.0 | 2.55 | 29.15 | 30.78 | 2 | 3 | 86.33 | 72.83 | 73.65 | 62.18 | 58.05 | 48.84 | 177.47 | 127.65 |
| RIL69 | 0.73 | 0.74 | 34.73 | 24.42 | 0.33 | 0.21 | 7.48 | 5.75 | 30.47 | 31.47 | 3 | 4 | 86.83 | 79.67 | 107.37 | 101.76 | 79.47 | 70.29 | 269.71 | 215.55 |
| RIL70 | 0.74 | 0.62 | 50.92 | 22.57 | 0.42 | 0.22 | 10.04 | 5.64 | 29.75 | 32.15 | 2 | 3 | 92.83 | 80.83 | 122.92 | 118.26 | 86.04 | 64.98 | 273.90 | 230.66 |
| RIL71 | 0.77 | 0.67 | 30.18 | 20.75 | 0.29 | 0.23 | 7.31 | 5.93 | 29.88 | 32.13 | 3 | 3 | 89.67 | 74.83 | 112.55 | 86.67 | 88.70 | 52.32 | 238.68 | 220.82 |
| RIL72 | 0.81 | 0.75 | 44.58 | 22.12 | 0.4 | 0.21 | 9.72 | 5.11 | 30.59 | 30.74 | 3 | 4 | 83.33 | 76.33 | 102.28 | 83.81 | 75.49 | 43.85 | 249.30 | 172.92 |
| RIL73 | 0.77 | 0.66 | 40.75 | 23.72 | 0.39 | 0.18 | 9.69 | 4.98 | 29.77 | 32.08 | 3 | 3 | 103.50 | 78.33 | 100.10 | 75.24 | 80.17 | 50.77 | 272.65 | 215.39 |
| RIL74 | 0.81 | 0.72 | 41.48 | 26.7 | 0.3 | 0.21 | 6.49 | 4.92 | 28.72 | 28.85 | 2 | 2 | 98.00 | 80.17 | 72.28 | 64.83 | 59.61 | 37.10 | 177.81 | 153.74 |
| RIL75 | 0.77 | 0.74 | 28.82 | 11.87 | 0.24 | 0.15 | 6.47 | 3.94 | 29.79 | 31.54 | 4 | 3 | 93.33 | 78.33 | 88.28 | 73.46 | 66.85 | 62.92 | 223.94 | 170.70 |
| RIL76 | 0.78 | 0.75 | 35.98 | 12.35 | 0.3 | 0.12 | 7.4 | 2.61 | 29.68 | 32.07 | 2 | 3 | 97.67 | 73.67 | 111.64 | 95.28 | 83.16 | 59.63 | 259.45 | 186.20 |
| RIL77 | 0.8 | 0.74 | 43.07 | 21.48 | 0.43 | 0.23 | 9.77 | 6.59 | 30.02 | 32.4 | 3 | 3 | 82.83 | 66.00 | 89.50 | 76.84 | 67.37 | 53.39 | 252.89 | 179.25 |
| RIL78 | 0.68 | 0.6 | 31.13 | 16.65 | 0.24 | 0.19 | 5.82 | 4.89 | 29.17 | 30.83 | 3 | 3 | 60.33 | 56.33 | 60.88 | 53.80 | 58.30 | 35.09 | 181.08 | 148.93 |
| RIL79 | 0.8 | 0.75 | 41.18 | 20.95 | 0.31 | 0.2 | 7.98 | 5.62 | 30.01 | 31.97 | 3 | 2 | 95.33 | 82.00 | 92.93 | 69.75 | 65.55 | 42.68 | 233.21 | 166.65 |
| RIL_Max | 0.83 | 0.77 | 50.92 | 31.23 | 0.48 | 0.42 | 11.33 | 9.33 | 30.89 | 34.03 | 3.5 | 3.83 | 106.00 | 92.50 | 137.17 | 118.26 | 102.12 | 75.61 | 329.20 | 246.57 |
| RIL_Min | 0.66 | 0.49 | 24.5 | 9.0 | 0.19 | 0.11 | 4.56 | 2.17 | 28.46 | 28.85 | 2 | 1.83 | 60.33 | 47.17 | 51.66 | 44.50 | 34.79 | 21.81 | 137.38 | 115.72 |
| RIL_AVG | 0.76 | 0.67 | 37.57 | 18.65 | 0.33 | 0.19 | 7.71 | 5.07 | 29.69 | 31.36 | 2.69 | 2.8 | 85.05 | 70.59 | 94.89 | 75.83 | 69.65 | 47.81 | 222.36 | 169.27 |
| CV% | 3.09 | 3.34 | 5.8 | 10.07 | 9.42 | 13.33 | 7.73 | 12.82 | 0.4 | 0.28 | 23.46 | 23.03 | 11.24 | 7.33 | 14.95 | 19.8 | 22.02 | 30.15 | 16.41 | 22.47 |
| LSD | 0.03 | 0.03 | 2.48 | 2.13 | 0.04 | 0.03 | 0.68 | 0.74 | 0.14 | 0.1 | 0.72 | 0.73 | 10.86 | 5.88 | 16.12 | 17.06 | 17.43 | 16.38 | 41.45 | 43.21 |
| SEd | 0.01 | 0.01 | 0.89 | 0.77 | 0.01 | 0.01 | 0.24 | 0.27 | 0.05 | 0.04 | 0.26 | 0.26 | 3.9 | 2.11 | 5.79 | 6.13 | 6.26 | 5.89 | 14.9 | 15.53 |

*NDVI: Normalized difference vegetation index; A_net_: Net CO_2_ assimilation rate* (μmol CO_2_ m^-2^ s^-1^)*; g_s_: Stomatal conductance to water vapor* (mol m^-2^ s^-1^)*; TR: Transpiration rate* (mmol H_2_O m^-2^ s^-1^)*; LT: Leaf temperature (^o^C); ASI: Anthesis silking interval (days); CH: Cob height(cm); CW: Cob weight(g/cob); TB: Total biomass(g/plant); GY: Grain yield(g/plant); WW: Well-watered; WD: Water-deficit stress; CV%: Coefficient of variation %; LSD: Least significant difference; SEd: Standard error*

**Table S4 Significance levels of normal distribution for various morpho-physiological and yield related traits using Kolmogorov–Smirnov test**

| Trait | Well-watered | | | Water-deficit | | |
| --- | --- | --- | --- | --- | --- | --- |
|  | Statistic | df | Significance test | Statistic | df | Significance test |
| NDVI | 0.061 | 79 | 0.200* | 0.092 | 79 | 0.092 |
| A_net_ | 0.052 | 79 | 0.200* | 0.068 | 79 | 0.200* |
| g_s_ | 0.108 | 79 | 0.024 | 0.081 | 79 | 0.200* |
| TR | 0.116 | 79 | 0.010 | 0.062 | 79 | 0.200* |
| LT | 0.088 | 79 | 0.200* | 0.078 | 79 | 0.200* |
| ASI | 0.132 | 79 | 0.002 | 0.126 | 79 | 0.003 |
| CH | 0.049 | 79 | 0.200* | 0.057 | 79 | 0.200* |
| CW | 0.063 | 79 | 0.200* | 0.068 | 79 | 0.200* |
| GY | 0.062 | 79 | 0.200* | 0.077 | 79 | 0.200* |
| TB | 0.068 | 79 | 0.200* | 0.086 | 79 | 0.200* |

*NDVI: Normalized difference vegetation index; A_net_: Net CO_2_ assimilation rate* (μmol CO_2_ m^-2^ s^-1^)*; g_s_: Stomatal conductance to water vapor* (mol m^-2^ s^-1^)*; TR: Transpiration rate* (mmol H_2_O m^-2^ s^-1^)*; LT: Leaf temperature (^o^C); ASI: Anthesis silking interval (days); CH: Cob height(cm); CW: Cob weight(g/cob); TB: Total biomass(g/plant); GY: Grain yield(g/plant);*

**Table S5 Descriptive statistics of the various morpho-physiological and yield related traits in parents and RIL population across environments.**

| Trait | Treatment | SNJ201126 | HKI161 | RIL population | | | | | |
| --- | --- | --- | --- | --- | --- | --- | --- | --- | --- |
|  |  | Grand Mean | Grand Mean | Grand Mean | Range | CV (%) | Skewness | Kurtosis | H^2^ |
| NDVI | WW | 0.77 | 0.73 | 0.76 | 0.66 - 0.83 | 3.09 | -0.48 | -0.06 | 0.68 |
|  | WD | 0.65 | 0.62 | 0.67 | 0.49 - 0.77 | 3.33 | -0.69 | 0.17 | 0.71 |
| A_net_ | WW | 43.33 | 37.28 | 37.56 | 24.5 - 50.92 | 5.80 | -0.02 | -0.49 | 0.67 |
|  | WD | 16.73 | 12.69 | 18.64 | 9.00 - 31.23 | 10.07 | 0.10 | -0.71 | 0.63 |
| g_s_ | WW | 0.40 | 0.36 | 0.32 | 0.19 - 0.48 | 9.41 | 0.27 | -0.63 | 0.66 |
|  | WD | 0.33 | 0.29 | 0.19 | 0.11 - 0.42 | 13.30 | 1.35 | 4.10 | 0.68 |
| TR | WW | 13.93 | 11.87 | 7.71 | 4.56 - 11.33 | 7.73 | 0.35 | -0.52 | 0.66 |
|  | WD | 8.67 | 7.00 | 5.07 | 2.17 - 9.33 | 12.80 | 0.60 | 1.75 | 0.65 |
| LT | WW | 30.78 | 37.18 | 29.69 | 28.46 - 30.89 | 0.40 | -0.04 | -1.09 | 0.47 |
|  | WD | 32.32 | 36.55 | 31.36 | 28.85 - 34.03 | 0.28 | -0.03 | 0.94 | 0.65 |
| ASI | WW | 1.83 | 2.67 | 2.69 | 2.00 - 3.50 | 23.46 | 0.35 | -0.56 | 0.45 |
|  | WD | 3.00 | 2.00 | 2.80 | 1.83 - 3.83 | 23.03 | 0.06 | -0.82 | 0.59 |
| CH | WW | 81.67 | 66.50 | 85.05 | 60.33 - 106 | 11.23 | 0.00 | -0.37 | 0.55 |
|  | WD | 61.83 | 53.17 | 70.59 | 47.17 - 92.5 | 7.33 | 0.02 | -0.43 | 0.66 |
| CW | WW | 89.83 | 80.33 | 94.89 | 51.66 - 137.17 | 14.95 | -0.01 | -0.58 | 0.60 |
|  | WD | 80.00 | 62.00 | 75.82 | 44.5 - 118.26 | 19.79 | 0.34 | -0.21 | 0.55 |
| TB | WW | 140.83 | 134.50 | 222.36 | 137.38 - 329.2 | 16.41 | 0.30 | -0.11 | 0.57 |
|  | WD | 121.17 | 114.17 | 169.27 | 115.72 - 246.57 | 22.47 | 0.51 | -0.46 | 0.55 |
| GY | WW | 66.00 | 49.00 | 69.64 | 34.79 - 102.12 | 22.02 | 0.01 | -0.47 | 0.58 |
|  | WD | 40.83 | 33.67 | 47.80 | 21.81 - 75.61 | 30.15 | 0.35 | -0.29 | 0.51 |

*NDVI: Normalized difference vegetation index; A_net_: Net CO_2_ assimilation rate* (μmol CO_2_ m^-2^ s^-1^)*; g_s_: Stomatal conductance to water vapor* (mol m^-2^ s^-1^)*; TR: Transpiration rate* (mmol H_2_O m^-2^ s^-1^)*; LT: Leaf temperature (^o^C); ASI: Anthesis silking interval (days); CH: Cob height(cm); CW: Cob weight(g/cob); TB: Total biomass(g/plant); GY: Grain yield(g/plant); WW: Well- watered; WD: Water-deficit stress; CV: Coefficient of variation; H^2^: Heritability in broad sense*

**Table S6 Combined ANOVA of the various morpho-physiological and yield related traits evaluated over two seasons and two treatments**

| Source | DF | NDVI | A_net_ | g_s_ | TR | LT | ASI | CH | CW | TB | GY |
| --- | --- | --- | --- | --- | --- | --- | --- | --- | --- | --- | --- |
| Replication (Season) | 4 | 0.002 | 20.69 | 0.01** | 2.62** | 0.00 | 4.03** | 173.74** | 5445.51** | 70437.42** | 3220.94** |
| Season | 1 | 1.001** | 1047.17** | 0.25** | 14.42** | 1.37** | 31.21** | 479115.34** | 374.51** | 132445.46** | 5921.25** |
| Treatment | 1 | 1.923** | 84847.69** | 4.39** | 1653.14** | 663.63** | 2.64 | 49525.22** | 86143.77** | 668050.05** | 113007.81** |
| Genotype | 78 | 0.024** | 214.88** | 0.03** | 12.76** | 4.76** | 1.24** | 909.24** | 3395.86** | 869895.18** | 1621.40** |
| Treatment × Genotype | 78 | 0.009** | 139.09** | 0.02** | 10.43** | 1.96** | 0.66 | 210.38** | 334.23** | 248393.48 | 415.00** |
| Season × Treatment | 1 | 0.036** | 5305.36** | 0.03** | 164.54** | 9.30** | 2.43* | 10480.07** | 2525.30** | 5060.29 | 2244.24** |
| Season × Genotype | 78 | 0.017** | 187.74** | 0.02** | 13.94** | 6.50** | 0.87** | 946.36** | 4103.91** | 977464.38** | 1902.71** |
| Season × Treatment × Genotype | 78 | 0.009** | 168.65** | 0.02** | 9.00** | 3.79** | 0.65 | 297.83** | 361.04** | 264937.64** | 345.87** |
| Error | 628 | 0.0005 | 4.34 | 0.0008 | 0.39 | 0.01 | 0.54 | 58.7 | 212.4 | 1384.94 | 220.18 |
| GM |  | 0.72 | 28.11 | 0.26 | 6.39 | 30.53 | 2.74 | 77.82 | 85.36 | 195.81 | 58.73 |
| CV% |  | 3.27 | 7.42 | 10.94 | 9.77 | 0.35 | 26.88 | 9.85 | 17.08 | 19.01 | 25.30 |
| CD_Trt_ |  | 0.00 | 0.27 | 0.00 | 0.08 | 0.01 | 0.09 | 0.98 | 1.86 | 4.75 | 1.90 |
| CD_Genotype_ |  | 0.02 | 1.67 | 0.02 | 0.50 | 0.08 | 0.59 | 6.15 | 11.69 | 29.84 | 11.91 |

**, ** represent values significant at 5% and 1% level respectively*

*NDVI: Normalized difference vegetation index; A_net_: Net CO_2_ assimilation rate* (μmol CO_2_ m^-2^ s^-1^)*; g_s_: Stomatal conductance to water vapor* (mol m^-2^ s^-1^)*; TR: Transpiration rate* (mmol H_2_O m^-2^ s^-1^)*; LT: Leaf temperature (^o^C); ASI: Anthesis silking interval (days); CH: Cob height(cm); CW: Cob weight(g/cob); TB: Total biomass(g/plant); GY: Grain yield(g/plant); Number of replications: 3; Seasons: 2; Treatments: 2, GM: Grand mean, CV%: Coefficient of variation, CD_Trt_: Critical difference of treatment at 5% level of significance; CD_Genotype_: Critical difference of genotype at 5% level of significance*

**Table S7 Correlations between traits in the RIL population in two seasons and two environments**

| Traits | NDVI | A_net_ | g_s_ | TR | LT | CH | CW | GY | TB | ASI |
| --- | --- | --- | --- | --- | --- | --- | --- | --- | --- | --- |
| NDVI | 1.00 | 0.12 | 0.25^*^ | 0.27^*^ | 0.14 | 0.35^**^ | 0.25^*^ | 0.23^*^ | 0.24^*^ | -0.17 |
| A_net_ | -0.13 | 1.00 | 0.56^**^ | 0.51^**^ | -0.12 | 0.14 | 0.10 | 0.10 | 0.06 | 0.06 |
| g_s_ | -0.13 | 0.63^**^ | 1.00 | 0.91^**^ | 0.18 | -0.03 | 0.34^**^ | 0.33^**^ | 0.16 | 0.05 |
| TR | -0.11 | 0.69^**^ | 0.94^**^ | 1.00 | 0.39^**^ | 0.00 | 0.39^**^ | 0.36^**^ | 0.24^*^ | 0.04 |
| LT | 0.30^**^ | -0.16 | -0.20 | -0.07 | 1.00 | -0.03 | 0.36^**^ | 0.27^*^ | 0.34^**^ | -0.06 |
| CH | 0.40^**^ | 0.11 | -0.11 | -0.08 | -0.06 | 1.00 | 0.09 | 0.13 | 0.23^*^ | -0.23^*^ |
| CW | 0.24^*^ | 0.00 | 0.17 | 0.17 | 0.02 | 0.31^**^ | 1.00 | 0.90^**^ | 0.68^**^ | -0.05 |
| GY | 0.3^**^ | 0.06 | 0.17 | 0.19 | 0.03 | 0.25^*^ | 0.71^**^ | 1.00 | 0.68^**^ | -0.06 |
| TB | 0.32^**^ | 0.14 | 0.08 | 0.16 | 0.11 | 0.4^**^ | 0.63^**^ | 0.69^**^ | 1.00 | -0.14 |
| ASI | 0.12 | 0.02 | 0.05 | 0.03 | 0.13 | -0.09 | 0.00 | 0.08 | 0.04 | 1.00 |

*Upper diagonal well-watered; lower diagonal water-deficit stress, *,** = test of significance at 5% and 1% level respectively at n-2 df*

*NDVI: Normalized difference vegetation index; A_net_: Net CO_2_ assimilation rate* (μmol CO_2_ m^-2^ s^-1^)*; g_s_: Stomatal conductance to water vapor* (mol m^-2^ s^-1^)*; TR: Transpiration rate* (mmol H_2_O m^-2^ s^-1^)*; LT: Leaf temperature (^o^C); ASI: Anthesis silking interval (days); CH: Cob height(cm); CW: Cob weight(g/cob); TB: Total biomass(g/plant); GY: Grain yield(g/plant);*

**Table S8 QTLs identified for various morpho-physiological and yield related traits under well-watered conditions**

| QTL name | Treatment | Chr | QTL type | Position of the QTL | Left Marker | Right Marker | LOD | PVE (%) | Add | Interval map distance (cM) |
| --- | --- | --- | --- | --- | --- | --- | --- | --- | --- | --- |
| *qA_net_3-2* | WW | 3 | Major | 324 | S3_174361072 | rs129386882 | 3.24 | 17.24 | -2.6 | 322.5 - 327.5 |
| *qg_s_6-2* | WW | 6 | Major | 271 | S6_89546385 | S6_179562549 | 2.54 | 15.86 | 0.03 | 252.5 - 282.5 |
| *qg_s_7-1* | WW | 7 | Minor | 162 | S7_166501967 | rs130671858 | 2.73 | 11.31 | 0.03 | 159.5 - 162.5 |
| *qTR1-1* | WW | 1 | Minor | 122 | rs128441140 | rs128842621 | 2.87 | 10.81 | 0.56 | 114.5 - 131.5 |
| *qTR3-1* | WW | 3 | Minor | 320 | rs129542985 | S3_173443508 | 2.72 | 10.11 | -0.55 | 319.5 - 320.5 |
| *qTR7-1* | WW | 7 | Major | 162 | S7_166501967 | rs130671858 | 6.07 | 21.47 | 0.78 | 160.5 - 162.5 |
| *qLT6-1* | WW | 6 | Minor | 246 | rs132369794 | S6_89546385 | 2.84 | 6.83 | 0.52 | 239.5 - 251.5 |
| *qLT8-1* | WW | 8 | Minor | 470 | S8_99035428 | S8_176308695 | 2.54 | 6.75 | -0.52 | 461.5 - 478.5 |
| *qASI1-2* | WW | 1 | Minor | 784 | rs131202973 | S1_10907875 | 3.02 | 14.91 | -0.15 | 771.5 - 791.5 |
| *qASI6-1* | WW | 6 | Minor | 6 | rs130449381 | S6_176162799 | 3.2 | 13.56 | 0.15 | 2.5 - 12.5 |
| *qCH8-1* | WW | 8 | Minor | 180 | rs130916094 | S8_165515995 | 2.77 | 14.9 | -3.65 | 177.5 - 181.5 |
| *qTB2-1* | WW | 2 | Minor | 445 | S2_213060766 | S2_18995588 | 2.53 | 13.51 | 14.79 | 439.5 - 458.5 |
| *qTB5-1* | WW | 5 | Minor | 70 | S5_26476971 | rs132230980 | 2.55 | 13.04 | 20.37 | 63.5 - 72.5 |

*A_net_: Net CO_2_ assimilation rate* (μmol CO_2_ m^-2^ s^-1^)*; g_s_: Stomatal conductance to water vapor* (mol m^-2^ s^-1^)*; TR: Transpiration rate* (mmol H_2_O m^-2^ s^-1^)*; LT: Leaf temperature (^o^C); ASI: Anthesis silking interval (days); CH: Cob height(cm); TB: Total biomass(g/plant); GY: Grain yield(g/plant); WW: Well-watered; WD: Water –deficit stress; LOD: Logarithm of odds ratio; PVE%: Total phenotypic variation in percentage explained by the QTL, Add: the additive effect of each QTL*

**Table S9 QTL × E interaction in RIL population over two seasons (*rainy and post rainy*)**

| Trait | Detection method* | chr | Posi-  tion | LeftMarker | RightMarker | LOD | LOD  (A) | LOD  (AbyE) | PVE | PVE  (A) | PVE  (AbyE) | Add | AbyE_01 | AbyE_02 | Left  CI | Right  CI |
| --- | --- | --- | --- | --- | --- | --- | --- | --- | --- | --- | --- | --- | --- | --- | --- | --- |
| A_net_ | ICIM-ADD, MET | 3 | 314 | S3_169283017 | S3_173528165 | 2.55 | 1.46 | 1.10 | 9.51 | 5.96 | 3.55 | -1.09 | 0.84 | -0.84 | 312.5 | 317.5 |
| A_net_ | ICIM-ADD, MET | 3 | 324 | S3_174361072 | rs129386882 | 3.23 | 1.89 | 1.35 | 15.53 | 7.61 | 7.92 | -1.22 | -1.24 | 1.24 | 322.5 | 327.5 |
| ASI | ICIM-ADD, MET | 1 | 786 | rs131202973 | S1_10907875 | 3.18 | 2.12 | 1.06 | 5.24 | 4.87 | 0.37 | -0.09 | -0.02 | 0.02 | 772.5 | 791.5 |
| ASI | ICIM-ADD, MET | 6 | 6 | rs130449381 | S6_176162799 | 3.87 | 2.89 | 0.98 | 7.45 | 7.26 | 0.18 | 0.12 | 0.02 | -0.02 | 2.5 | 9.5 |
| CH | ICIM-ADD, MET | 1 | 266 | S1_38965222 | S1_38965211 | 5.42 | 5.39 | 0.04 | 11.94 | 11.94 | 0.00 | 3.49 | 0.03 | -0.03 | 263.5 | 266.5 |
| CH | ICIM-ADD, MET | 1 | 740 | S1_6365045 | rs818095140 | 5.41 | 5.31 | 0.10 | 11.30 | 11.10 | 0.20 | 3.54 | -0.47 | 0.47 | 738.5 | 741.5 |
| CH | ICIM-ADD, MET | 3 | 235 | S3_194753667 | rs277236564 | 4.07 | 3.87 | 0.20 | 8.04 | 7.95 | 0.09 | -2.79 | 0.29 | -0.29 | 232.5 | 236.5 |
| CH | ICIM-ADD, MET | 8 | 180 | rs130916094 | S8_165515995 | 3.19 | 2.85 | 0.34 | 7.36 | 5.92 | 1.43 | -2.44 | -1.20 | 1.20 | 177.5 | 181.5 |
| CW | ICIM-ADD, MET | 2 | 443 | S2_213060766 | S2_18995588 | 7.23 | 6.96 | 0.27 | 13.78 | 13.75 | 0.03 | 8.00 | 0.39 | -0.39 | 437.5 | 445.5 |
| g_s_ | ICIM-ADD, MET | 3 | 168 | S3_5950551 | S3_5721251 | 3.33 | 1.16 | 2.17 | 6.14 | 3.50 | 2.64 | -0.01 | 0.01 | -0.01 | 164.5 | 171.5 |
| g_s_ | ICIM-ADD, MET | 6 | 109 | S6_126753475 | rs836167502 | 4.66 | 2.51 | 2.15 | 10.17 | 8.36 | 1.81 | -0.02 | 0.01 | -0.01 | 106.5 | 112.5 |
| g_s_ | ICIM-ADD, MET | 7 | 162 | S7_166501967 | rs130671858 | 3.53 | 3.53 | 0.00 | 15.53 | 12.25 | 3.28 | 0.02 | 0.01 | -0.01 | 159.5 | 162.5 |
| g_s_ | ICIM-ADD, MET | 7 | 372 | S7_139259301 | S7_139259336 | 2.89 | 0.75 | 2.14 | 5.71 | 2.64 | 3.06 | 0.01 | -0.01 | 0.01 | 370.5 | 374.5 |
| GY | ICIM-ADD, MET | 2 | 454 | S2_18995588 | S2_14679066 | 4.30 | 4.12 | 0.18 | 8.97 | 8.93 | 0.04 | 4.74 | 0.31 | -0.31 | 445.5 | 462.5 |
| GY | ICIM-ADD, MET | 6 | 267 | S6_89546385 | S6_179562549 | 3.71 | 3.53 | 0.17 | 7.49 | 7.46 | 0.04 | 4.31 | 0.31 | -0.31 | 263.5 | 278.5 |
| NDVI | ICIM-ADD, MET | 2 | 174 | rs812099243 | rs822182360 | 3.97 | 3.91 | 0.06 | 13.55 | 10.94 | 2.61 | -0.02 | 0.01 | -0.01 | 172.5 | 175.5 |
| NDVI | ICIM-ADD, MET | 2 | 389 | rs131350195 | S2_66658066 | 5.01 | 4.99 | 0.03 | 16.96 | 13.89 | 3.07 | 0.02 | -0.01 | 0.01 | 381.5 | 390.5 |
| TB | ICIM-ADD, MET | 2 | 449 | S2_18995588 | S2_14679066 | 4.32 | 4.31 | 0.01 | 11.90 | 11.66 | 0.24 | 12.28 | 1.76 | -1.76 | 441.5 | 459.5 |
| TR | ICIM-ADD, MET | 1 | 121 | rs128441140 | rs128842621 | 3.98 | 3.86 | 0.12 | 11.36 | 10.50 | 0.86 | 0.39 | 0.11 | -0.11 | 114.5 | 127.5 |
| TR | ICIM-ADD, MET | 3 | 320 | rs129542985 | S3_173443508 | 3.17 | 2.82 | 0.35 | 10.12 | 8.31 | 1.81 | -0.36 | -0.17 | 0.17 | 319.5 | 320.5 |
| TR | ICIM-ADD, MET | 7 | 162 | S7_166501967 | rs130671858 | 6.29 | 4.81 | 1.48 | 21.70 | 14.36 | 7.34 | 0.45 | 0.32 | -0.32 | 160.5 | 162.5 |
| A_net_ | MET | 5 | 71 | S5_26476971 | rs132230980 | 2.72 | 2.58 | 0.14 | 10.22 | 10.14 | 0.08 | -1.90 | 0.17 | -0.17 | 65.5 | 76.5 |
| ASI | MET | 1 | 600 | S1_59613063 | S1_51302779 | 2.66 | 2.64 | 0.02 | 6.71 | 6.01 | 0.70 | -0.10 | 0.04 | -0.04 | 589.5 | 606.5 |
| ASI | MET | 4 | 39 | S4_216350824 | S4_24592607 | 2.72 | 2.70 | 0.02 | 7.08 | 6.34 | 0.74 | 0.13 | -0.05 | 0.05 | 33.5 | 42.5 |
| ASI | MET | 7 | 514 | S7_4714364 | rs810411622 | 2.71 | 2.14 | 0.57 | 5.08 | 5.07 | 0.00 | 0.09 | 0.00 | 0.00 | 509.5 | 516.5 |
| CH | MET | 5 | 504 | S5_196467650 | S5_193365346 | 2.51 | 2.45 | 0.06 | 5.02 | 5.01 | 0.01 | 2.19 | -0.10 | 0.10 | 500.5 | 508.5 |
| CH | MET | 6 | 325 | S6_178475216 | rs278133365 | 2.83 | 2.42 | 0.41 | 5.23 | 4.89 | 0.34 | 2.65 | -0.70 | 0.70 | 317.5 | 330.5 |
| CW | MET | 2 | 102 | S2_235239563 | S2_16162881 | 3.11 | 3.11 | 0.01 | 6.34 | 6.13 | 0.21 | 5.31 | 0.99 | -0.99 | 96.5 | 111.5 |
| CW | MET | 4 | 36 | S4_216350824 | S4_24592607 | 2.70 | 2.70 | 0.00 | 5.88 | 5.67 | 0.21 | 6.28 | 1.20 | -1.20 | 28.5 | 38.5 |
| CW | MET | 4 | 319 | rs129956711 | rs825739889 | 2.71 | 2.59 | 0.13 | 5.03 | 5.02 | 0.01 | 4.74 | 0.25 | -0.25 | 311.5 | 327.5 |
| CW | MET | 5 | 400 | S5_183503547 | rs130161307 | 2.60 | 2.22 | 0.38 | 3.93 | 3.89 | 0.05 | 4.18 | -0.46 | 0.46 | 390.5 | 410.5 |
| CW | MET | 6 | 258 | rs132369794 | S6_89546385 | 3.82 | 3.79 | 0.03 | 7.98 | 7.82 | 0.16 | 5.94 | 0.84 | -0.84 | 247.5 | 262.5 |
| CW | MET | 7 | 335 | S7_136261075 | rs821069694 | 3.39 | 3.34 | 0.04 | 5.72 | 5.63 | 0.09 | -5.16 | -0.65 | 0.65 | 329.5 | 337.5 |
| g_s_ | MET | 1 | 121 | rs128441140 | rs128842621 | 4.26 | 3.91 | 0.35 | 12.13 | 11.65 | 0.48 | 0.02 | 0.00 | 0.00 | 113.5 | 127.5 |
| g_s_ | MET | 5 | 91 | S5_213695835 | S5_220103695 | 2.64 | 2.63 | 0.01 | 10.01 | 8.18 | 1.84 | -0.02 | -0.01 | 0.01 | 87.5 | 103.5 |
| g_s_ | MET | 8 | 232 | S8_152991912 | S8_123369436 | 2.65 | 0.00 | 2.65 | 5.59 | 0.13 | 5.46 | 0.00 | -0.01 | 0.01 | 225.5 | 243.5 |
| GY | MET | 2 | 172 | S2_241498576 | rs812099243 | 2.78 | 2.69 | 0.09 | 5.20 | 5.15 | 0.06 | -3.61 | -0.38 | 0.38 | 170.5 | 173.5 |
| GY | MET | 4 | 320 | rs129956711 | rs825739889 | 3.26 | 3.25 | 0.01 | 6.71 | 6.49 | 0.22 | 4.00 | 0.74 | -0.74 | 314.5 | 327.5 |
| GY | MET | 7 | 184 | rs815472538 | rs807579269 | 3.18 | 2.93 | 0.26 | 5.96 | 5.96 | 0.00 | 3.97 | 0.00 | 0.00 | 170.5 | 194.5 |
| LT | MET | 1 | 210 | S1_87218665 | rs128649603 | 2.82 | 2.82 | 0.01 | 6.45 | 6.21 | 0.24 | 0.21 | -0.04 | 0.04 | 205.5 | 212.5 |
| LT | MET | 5 | 70 | S5_26476971 | rs132230980 | 4.19 | 4.15 | 0.04 | 9.11 | 8.99 | 0.12 | 0.37 | -0.04 | 0.04 | 66.5 | 72.5 |
| LT | MET | 6 | 229 | rs726897229 | rs278162328 | 3.22 | 3.11 | 0.11 | 6.82 | 6.80 | 0.02 | 0.22 | -0.01 | 0.01 | 215.5 | 231.5 |
| LT | MET | 7 | 437 | S7_175880407 | rs130683859 | 2.98 | 2.94 | 0.04 | 7.10 | 6.41 | 0.69 | 0.22 | -0.07 | 0.07 | 436.5 | 438.5 |
| LT | MET | 8 | 424 | S8_68442605 | S8_99035421 | 2.73 | 2.58 | 0.15 | 5.73 | 5.73 | 0.00 | -0.21 | 0.00 | 0.00 | 416.5 | 437.5 |
| NDVI | MET | 3 | 222 | rs831045561 | rs129375826 | 3.82 | 3.70 | 0.12 | 9.46 | 9.24 | 0.23 | -0.02 | 0.00 | 0.00 | 217.5 | 224.5 |
| NDVI | MET | 3 | 486 | S3_32130689 | S3_127383531 | 2.55 | 1.60 | 0.95 | 4.71 | 4.12 | 0.60 | 0.01 | 0.00 | 0.00 | 483.5 | 493.5 |
| NDVI | MET | 7 | 235 | S7_31370165 | S7_9453507 | 2.88 | 2.88 | 0.00 | 7.87 | 7.22 | 0.64 | -0.02 | 0.01 | -0.01 | 233.5 | 239.5 |
| NDVI | MET | 8 | 83 | S8_178117997 | S8_177342722 | 2.64 | 2.24 | 0.40 | 5.79 | 5.77 | 0.02 | -0.01 | 0.00 | 0.00 | 79.5 | 85.5 |
| NDVI | MET | 10 | 228 | rs830243764 | rs128480638 | 2.51 | 2.47 | 0.04 | 6.93 | 6.07 | 0.86 | -0.01 | 0.00 | 0.00 | 224.5 | 237.5 |
| TB | MET | 3 | 143 | S3_3911409 | rs129396541 | 2.52 | 2.51 | 0.01 | 6.22 | 6.15 | 0.07 | 11.96 | 1.28 | -1.28 | 138.5 | 147.5 |
| TB | MET | 5 | 69 | S5_26476971 | rs132230980 | 2.71 | 2.21 | 0.50 | 7.01 | 5.27 | 1.75 | 11.61 | 6.69 | -6.69 | 63.5 | 72.5 |
| TB | MET | 6 | 329 | S6_178475216 | rs278133365 | 3.16 | 2.91 | 0.26 | 7.28 | 7.16 | 0.12 | 11.24 | -1.45 | 1.45 | 322.5 | 335.5 |
| TB | MET | 7 | 431 | S7_170821775 | rs130680787 | 2.59 | 2.53 | 0.06 | 6.77 | 6.35 | 0.43 | 8.98 | 2.32 | -2.32 | 428.5 | 434.5 |

**Some of the QTLs were identified by MET (multi environmental trials) module were also identified by ICIM-ADD method.*

*NDVI: Normalized difference vegetation index; A_net_: Net CO_2_ assimilation rate* (μmol CO_2_ m^-2^ s^-1^)*; g_s_: Stomatal conductance to water vapor* (mol m^-2^ s^-1^)*; TR: Transpiration rate* (mmol H_2_O m^-2^ s^-1^)*; LT: Leaf temperature (^o^C); ASI: Anthesis silking interval (days); CH: Cob height(cm); CW: Cob weight(g/cob); TB: Total biomass(g/plant); GY: Grain yield(g/plant); LOD: Logarithm of odds ratio for all effects; LOD(A): LOD score for additive and dominance effects; LOD (AbyE)-LOD score for additive and dominance by environment effects; PVE%: Phenotypic variation explained by all effects; PVE(A)-Phenotypic variation explained by additive and dominance effects; PVE (AbyE)-Phenotypic variation explained by additive and dominance by environment effect at the current scanning position, AbyE_01-Additive and dominance by environment 1 effect at the current scanning position; AbyE_02-Additive and dominance by environment 2 effect at the current scanning position*

**Table S10 List of annotated genes present within the QTLs identified under well-watered conditions for various morpho-physiological and yield traits**

| **QTL name** | **chr** | **Position**  **(start - end bp)** | **Position of SNP** | **SNP** | **Gene size (bp)** | **Locus ID** | **Annotation** | **Biological process** |
| --- | --- | --- | --- | --- | --- | --- | --- | --- |
| *qA_net_3-2* | 3 | 177560743-177565229 | 177563598 | T | 4112 | *Zm00001eb146040* | Chloride channel protein | Chloride transmembrane transporter activity |
| *qg_s_6-2* | 6 | 179555644-179566050 | 179562549 | C | 7615 | *Zm00001eb297570* | Protein-serine/threonine phosphatase | Protein dephosphorylation |
|  | 6 | 179563176-179566051 | 179562549 | C | 2875 | *Zm00001eb297580* | Penta-tricopeptide repeat-containing protein mitochondrial | Zinc ion binding |
| *qg_s_7-1* | 7 | 166501156-166503762 | 166501967 | G | 2606 | *Zm00001eb324180* | Sugar carrier protein C | Symporter activity |
|  | 7 | 166624336-166625597 | 166627700 | T | 1261 | *Zm00001eb324240* | Chlorophyll a-b binding protein, chloroplastic | Photosynthesis, light harvesting in photosystem I |
| *qTR1-1* | 1 | 53207544-53208672 | 53209688 | C | 1128 | *Zm00001eb015510* | Phospholipid-transporting ATPase | Phospholipid transport |
| *qTR3-1* | 3 | 173658878-173662031 | 173660957 | A | 3153 | *Zm00001eb145080* | Pectin acetylesterase | Cell wall organization |
|  | 3 | 173440780-173444468 | 173443508 | C | 3688 | *Zm00001eb144960* | Lipoxygenase | Cell wall and cell membrane biosynthetic process |
| *qTR7-1* | 7 | 166501156-166503762 | 166501967 | G | 2606 | *Zm00001eb324180* | Sugar carrier protein C | Symporter activity |
|  | 7 | 166624336-166625597 | 166627700 | T | 1261 | *Zm00001eb324240* | Chlorophyll a-b binding protein, chloroplastic | Photosynthesis, light harvesting in photosystem I |
| *qLT6-1* | 6 | 175617292-175620402 | 175623889 | G | 3110 | *Zm00001eb295640* | Rop guanine nucleotide exchange factor 9 | Guanyl-nucleotide exchange factor activity |
|  | 6 | 175623148-175625984 | 175623889 | G | 2799 | *Zm00001eb295670* | Putative leucine-rich repeat transmembrane protein kinase family protein | Protein kinase activity |
| *qLT8-1* | 8 | 176300393-176321965 | 176308695 | G | 21572 | *Zm00001eb368460* | Outer membrane OMP85 family protein | Cellular protein-containing complex assembly |
| *qASI1-2* | 1 | 7953030-7955203 | 7954575 | T | 2173 | *Zm00001eb002830* | bHLH-transcription factor 35 | Protein dimerization activity |
|  | 1 | 10907217-10908561 | 10907875 | G | 1344 | *Zm00001eb003910* | LOB transcription factor | Hormone-mediated signaling pathway |
| *qASI6-1* | 6 | 175953581-175955309 | 175957266 | G | 1728 | *Zm00001eb295810* | NAC type transcription factor (NAC87) | Regulation of transcription |
| *qCH8-1* | 8 | 168814976-168827925 | 168821861 | T | 12809 | *Zm00001eb364820* | AP-4 complex subunit epsilon | Intracellular protein transport |
|  | 8 | 165513419-165517693 | 165515995 | C | 4274 | *Zm00001eb363270* | Calcium-transporting ATPase | Calcium transport |
| *qTB2-1* | 2 | 213050055-213064093 | 213060766 | T | 4203 | *Zm00001eb107320* | Organic cation/carnitine transporter 7 | Symporter activity |
| *qTB5-1* | 5 | 26473449-26493353 | 26476971 | C | 19904 | *Zm00001eb221070* | Tetra-tricopeptide repeat (TPR)-like superfamily protein | Cell organelle biogenesis |
|  | 5 | 153353115-153354302 | 153354583 | C | 1187 | *Zm00001eb239390* | Meiosis 5 | Cell division |

*A_net_: Net CO_2_ assimilation rate* (μmol CO_2_ m^-2^ s^-1^)*; g_s_: Stomatal conductance to water vapor* (mol m^-2^ s^-1^)*; TR: Transpiration rate* (mmol H_2_O m^-2^ s^-1^)*; LT: Leaf temperature (^o^C); ASI: Anthesis silking interval (days); CH: Cob height(cm); TB: Total biomass(g/plant)*

**Table S11 The genetic, physical distance and number of annotated genes present within the QTLs regions identified for various morpho-physiological and yield traits under water deficit stress conditions**

| **QTL name** | **chr** | **Left Marker** | **Right Marker** | **QTL interval** | **Physical position_ start** | **Physical position_ end** | **Size (Mbp)** | **No. of genes** |
| --- | --- | --- | --- | --- | --- | --- | --- | --- |
| *qNDVI2-1* | 2 | rs812099243 | rs822182360 | 172.5 - 175.5 | 241930638 | 241835286 | 0.10 | 22 |
| *qNDVI2-2* | 2 | rs131350195 | S2_66658066 | 378.5 - 388.5 | 62951436 | 66658066 | 3.71 | 108 |
| *qA_net_3-1* | 3 | S3_169283017 | S3_173528165 | 312.5 - 317.5 | 169283017 | 173528165 | 4.25 | 264 |
| *qg_s_3-1* | 3 | S3_5950551 | S3_5721251 | 164.5 - 171.5 | 5950551 | 5721251 | 0.23 | 35 |
| *qg_s_6-1* | 6 | S6_126753475 | rs836167502 | 105.5 - 112.5 | 126753475 | 120736548 | 6.02 | Not ascertained |
| *qg_s_7-2* | 7 | S7_139259301 | S7_139259336 | 370.5 - 374.5 | 139259301 | 139259336 | 0.00 | 1 |
| *qASI1-1* | 1 | rs128441140 | rs128842621 | 120.5 - 139.5 | 53209688 | 204879452 | 151.67 | Not ascertained |
| *qCH1-1* | 1 | S1_38965222 | S1_38965211 | 263.5 - 266.5 | 38965222 | 38965211 | 0.00 | 1 |
| *qCH1-2* | 1 | S1_6365045 | rs818095140 | 738.5 - 741.5 | 6365045 | 6190530 | 0.17 | 31 |
| *qCH3-1* | 3 | S3_194753667 | rs277236564 | 232.5 - 239.5 | 194753667 | 195627269 | 0.87 | 64 |
| *qCW2-1* | 2 | S2_213060766 | S2_18995588 | 434.5 - 445.5 | 213060766 | 18995588 | 194.07 | Not ascertained |
| *qTB2-2, qGY2-1* | 2 | S2_18995588 | S2_14679066 | 444.5 - 464.5 | 18995588 | 14679066 | 4.32 | 372 |
| *qGY6-1* | 6 | S6_89546385 | S6_179562549 | 250.5 - 286.5 | 89546385 | 179562549 | 90.02 | Not ascertained |

*NDVI: Normalized difference vegetation index; A_net_: Net CO_2_ assimilation rate* (μmol CO_2_ m^-2^ s^-1^)*; g_s_: Stomatal conductance to water vapor* (mol m^-2^ s^-1^)*; ASI: Anthesis silking interval (days); CH: Cob height(cm); CW: Cob weight(g/cob); TB: Total biomass(g/plant); GY: Grain yield(g/plant);*

**TABLE S12 QTLs detected by other studies in the QTL regions identified in the present study**

| **QTL name** | **Other QTLs in the same region** | **Bin No.** | **Position (+ strand)** | **Reference allele** | **Alternate allele** | **RSID** | **Reference** |
| --- | --- | --- | --- | --- | --- | --- | --- |
| *A_net_3-1* | Chlorophyll content | *3.05* | 169275678..169275778 | G | C | rs132050466 | Yi et al. 2020 |
| *A_net_3-1* | Ear infructescence position | *3.06* | 173536095..173536195 | T | A | rs129572829 | Peiffer et al. 2014 |
| *A_net_3-1* | Leaf width | *3.06* | 173536670..173536770 | C | T | rs132054902 | Tian et al. 2010 |
| *qg_s_3-1* | The coefficients of variation of days to silking | *3.02* | 5719519..5719619 | C | G | rs276967931 | Li et al. 2016 |
| *qg_s_3-1* | The coefficients of variation of days to anthesis | *3.02* | 5721213..5721313 | NA | NA | NA | Li et al. 2016 |
| *qg_s_3-1* | Beta-cryptoxanthin content | *3.02* | 5721096..5721196 | NA | NA | NA | Li et al. 2016 |
| *qg_s_3-1* | Stalk bending strength | *3.02* | 5719654..5719754 | NA | NA | NA | Peiffer et al. 2014 |
| *qASI1-1* | Ear infructescence position | *1.04* | 53208334..53208434 | G | T | rs725492131 | Peiffer et al. 2014 |
| *qCH3-1* | Plant height | *3.06* | 195627612..195627712 | NA | NA | NA | Peiffer et al. 2014 |
| *qCH3-1* | Relative chlorophyll content | *3.06* | 195628298..195628398 | C | A | rs817540197 | Yi et al. 2020 |
| *qCH3-1* | Ear infructescence position | *3.06* | 195627612..195627712 | NA | NA | NA | Peiffer et al 2014 |
| *qGY6-1* | Ear diameter | *6.08* | 179562399..179562499 | NA | NA | NA | Kusmec et al. 2017 |
| *qGY6-1* | Kernel row number per ear | *6.08* | 179562984..179563084 | A | G | rs132377029 | Brown et al. 2011 |

*A_net_: Net CO_2_ assimilation rate* (μmol CO_2_ m^-2^ s^-1^)*; g_s_: Stomatal conductance to water vapor* (mol m^-2^ s^-1^)*; ASI: Anthesis silking interval (days); CH: Cob height(cm); GY: Grain yield(g/plant);*

**Supplementary Figures**

**Figure S1** Weekly weather data during the crop growth period (a) *Rainy season* 2018 (b) *Post rainy season* 2018-19

**
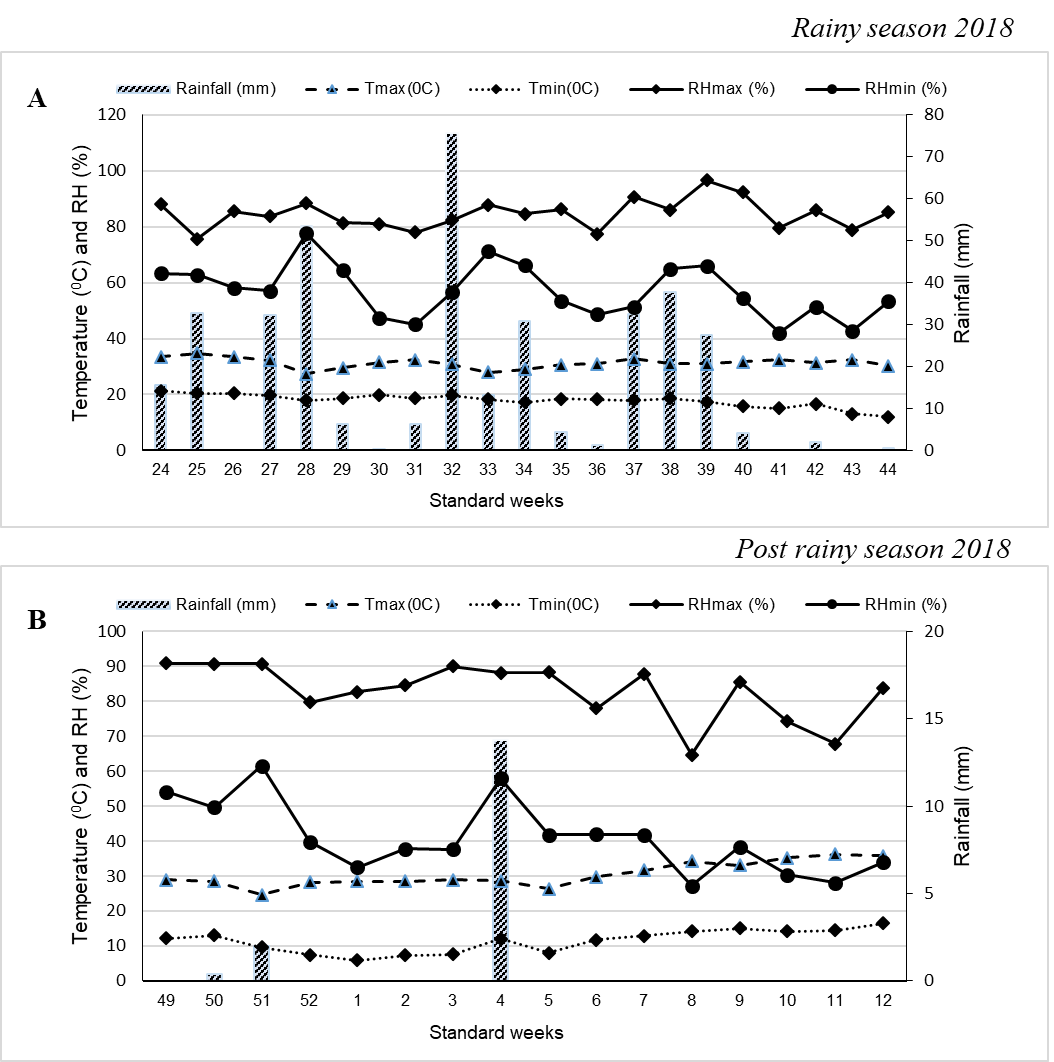
**

**Figure S2A** Significant major effect epistatic interactions identified for the traits net CO_2_ assimilation rate (A_net_), g_s_: Stomatal conductance to water vapor; TR: Transpiration rate; leaf temperature (LT), WW: well-watered; WD: water deficit stress.


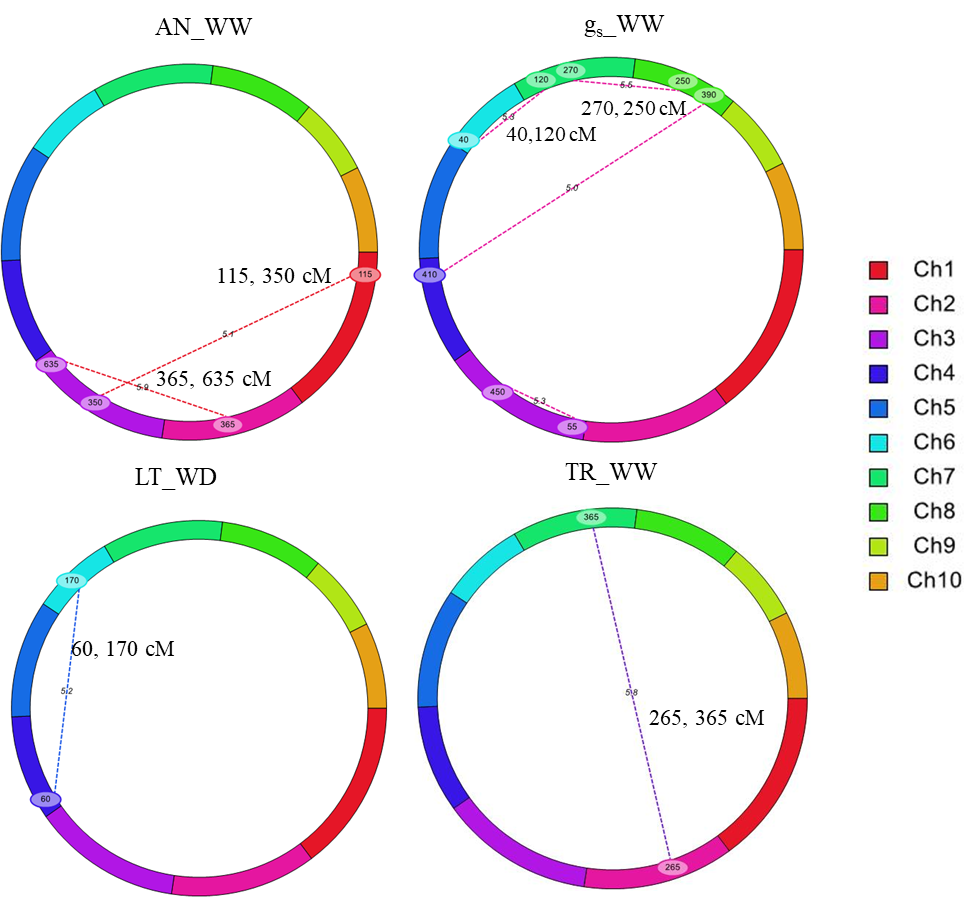


**Figure S2B** Significant major effect epistatic interactions identified for the traits anthesis-silking interval, cob weight (CW), total biomass (TB); WD: water-deficit stress


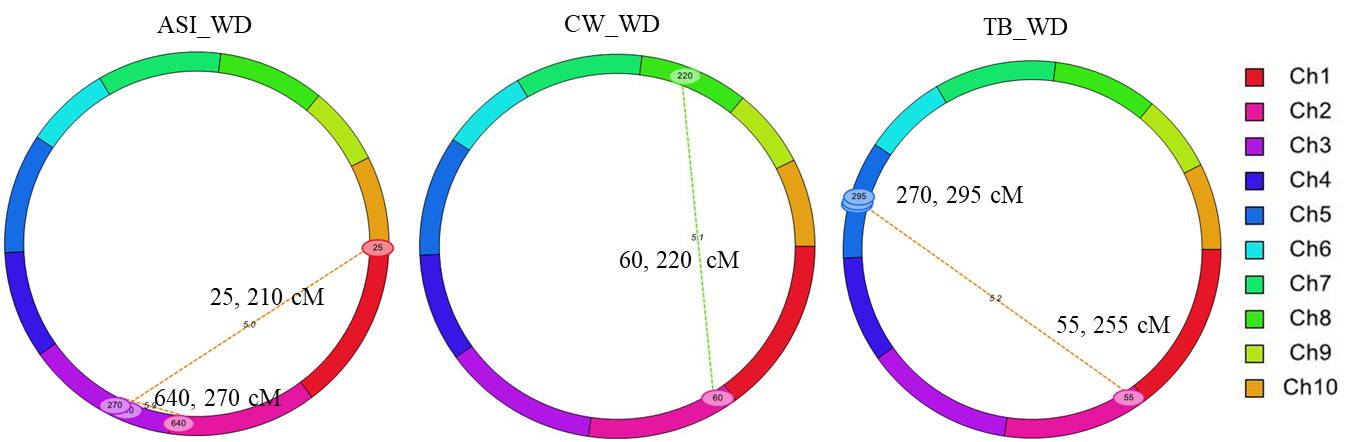

Supplement: Supplementary file 1 [file DataSheet_1.docx]
